# Comparative effects of atorvastatin and rosuvastatin on inflammatory biomarkers: a systematic review and meta-analysis of randomized head-to-head trials

**DOI:** 10.1186/s12872-026-06386-4

**Published:** 2026-09-10

**Authors:** Abu Omayer, Mohamed I. Mohamed, Maryam Shahid, Syed Mohammed Hassanul Hoque, Nour Elhuda Ahmed Mostafa Mohamed, Enjy Mohamed Amer Elsayed, Moussa Nassar, Tarique Ahmed, Yasar Sattar

**Affiliations:** 1 Texas Tech University Health Sciences Center (Permian Basin), Lubbock, TX USA; 2 Faculty of Medicine, Alexandria University, Alexandria, Egypt; 3 Aster Hospital, Al Qusais, Dubai, United Arab Emirates; 4 University of Georgia, Tbilisi, Georgia; 5 Gilbert and Rose-Marie Chagoury School of Medicine, Lebanese American University, Byblos, Lebanon; 6 Department of Cardiology, Cathay General Hospital, Taipei, Taiwan; 7 Department of Cardiology, West Virginia University, Morgantown, WV 26505 USA

**Keywords:** Atorvastatin, Rosuvastatin, C-reactive protein, Interleukin 6, Adiponectin, Tumor necrosis factor-alpha

## Abstract

**Background:**

Inflammation contributes significantly to cardiovascular disease progression. While both atorvastatin and rosuvastatin have anti-inflammatory effects, evidence directly comparing their effects on inflammatory biomarkers is limited and inconsistent. This study aimed to compare their effects through a meta-analysis of randomized controlled trials (RCTs).

**Methods:**

We performed a systematic review and meta-analysis of head-to-head RCTs comparing atorvastatin and rosuvastatin on inflammatory biomarkers. On August 14, 2024, we searched PubMed, Web of Science, Scopus, and CENTRAL, and subsequently searched ClinicalTrials.gov for unpublished studies. We included RCTs directly comparing the two statins and reported changes in C-reactive protein (CRP), interleukin-6 (IL-6), tumor necrosis factor-alpha (TNF-α), or adiponectin. Using a random-effects model, we pooled the data and reported the results as mean differences (MD) or standardized mean differences (SMD) with 95% confidence intervals (CIs). We assessed risk of bias using the Cochrane RoB 2 tool. The review protocol was registered in PROSPERO (CRD42024579712) (see the PRISMA 2020 for Abstracts checklist).

**Results:**

A total of 35 RCTs (*n* = 6,148) were included. The pooled effect showed no significant difference in CRP reduction (MD: − 0.53 mg/L; 95% CI: − 1.10 to 0.05; *p* = 0.07; I^2^ = 82%). After excluding one outlier, rosuvastatin showed a greater reduction (MD: − 0.16 mg/L; 95% CI: − 0.28 to − 0.03; *p* = 0.01; I^2^ = 63%). The SMD also favored rosuvastatin (SMD: − 0.17; 95% CI: − 0.31 to − 0.04; *p* = 0.01). Sensitivity analysis among low risk-of-bias studies (*n* = 9) showed a smaller yet significant reduction (MD: − 0.08 mg/L; 95% CI: − 0.16 to − 0.01; *p* = 0.03). We observed no significant differences for IL-6 (*n* = 484; MD: − 0.82; 95% CI: − 3.02 to 1.38), adiponectin (*n* = 257; MD: − 1.78; 95% CI: − 4.70 to 1.14), or TNF-alpha (*n* = 309; MD: 0.23; 95% CI: − 0.05 to 0.52). Meta-regression identified baseline CRP, HDL, and hypertension prevalence as predictors of CRP reduction.

**Conclusions:**

Rosuvastatin showed a modest but statistically significant advantage over atorvastatin in lowering CRP, especially in patients with higher baseline inflammation or hypertension. Evidence for other biomarkers remains inconclusive. Tailoring statin therapy to patient profiles may optimize benefits.

**Supplementary Information:**

The online version contains supplementary material available at https://doi.org/10.1186/s12872-026-06386-4.

## Introduction

Dyslipidemia, characterized by abnormal lipid levels, is a major atherosclerotic risk factor for cardiovascular diseases, contributing to morbidity and mortality [1]. Dyslipidemia can be due to underlying abnormal inflammatory markers, including C-reactive protein (CRP), interleukin-6 (IL-6), tumor necrosis factor-alpha (TNF-alpha), and adiponectin levels. Coronary artery disease (CAD) contributes to 7 million deaths and 129 million disability-adjusted life-years (DALYs) worldwide; outside the USA, CAD-related mortality can be as high as 19 million. According to the World Heart Federation, global CAD deaths increased from 12.1 million in 1990 to 20.5 million in 2021. In contrast, the overall cardiovascular disease mortality rate decreased from 354.5 per 100,000 in 1990 to 239.9 per 100,000 in 2019 [2, 3].

CAD poses a very high risk of future major adverse cardiovascular events (MACE), with a significant contribution due to high low-density lipoprotein (LDL) levels. Statins lower the levels of LDL in the blood by blocking 3-hydroxy-methylglutaryl coenzyme A (HMG-CoA) reductase, which is essential for the synthesis of cholesterol [4]. Statins, particularly rosuvastatin and atorvastatin, provide additional benefits through their anti-inflammatory effects on CRP and IL-6, alongside their lipid-lowering properties, which help manage dyslipidemia and CVD [5]. Managing inflammatory markers such as CRP, IL-6, TNF-alpha, and adiponectin in CAD patients is crucial, as elevated levels are linked to cardiac events, hypertension, atherosclerosis, and heart failure [6]. Elevated CRP in dyslipidemia patients increases the risk of comorbidities such as stroke [7]. High levels of IL-6 are linked to the development of arterial stiffness and microvascular dysfunction [8]. According to a 2016 survey conducted by the Korean National Health and Nutrition Examination, participants with cardiovascular illness had higher hs-CRP levels [9].

Previous meta-analyses comparing atorvastatin and rosuvastatin have reported conflicting findings regarding CRP reduction; one found no significant difference, while another favored rosuvastatin [10, 11]. However, both were limited to CRP and largely excluded patients with broader clinical conditions. In contrast, the present study aims to evaluate a broader spectrum of inflammatory biomarkers, including IL-6, TNF-alpha, and adiponectin. By incorporating patients with comorbidities such as dyslipidemia, diabetes, and acute coronary syndrome, our study aims to present the most comprehensive and clinically relevant assessment to date of the comparative anti-inflammatory effects of atorvastatin and rosuvastatin.

## Methods

The protocol for this review was registered in PROSPERO (CRD42024579712). The review was conducted and reported according to the Preferred Reporting Items for Systematic Reviews and Meta-Analyses (PRISMA) Guidelines [12]. The PRISMA 2020 checklist was completed and is available in Additional File 7.

A systematic search was conducted across four major bibliographic databases: PubMed, Web of Science, Scopus, and CENTRAL. This search was executed on August 14, 2024, using relevant terms such as “Atorvastatin,” “Rosuvastatin,” “C-reactive protein,” “Interleukin 6,” “Adiponectin,” and “Tumor necrosis factor-alpha.” Full search strategies are provided in Additional file 1. To ensure comprehensiveness, we re-applied the search strategy before final analysis and manually screened the reference lists of prior meta-analyses to identify additional eligible studies. After completing the primary review process, we also searched ClinicalTrials.gov to assess for any unpublished or missed studies. We did not search grey literature sources (e.g., OpenGrey or conference proceedings), and trial registry screening was limited to ClinicalTrials.gov.

The inclusion criteria centered on randomized controlled trials (RCTs) that compared two statin drugs: atorvastatin and rosuvastatin. RCTs were selected exclusively because they represent the highest level of clinical evidence, minimizing bias and confounding, whereas observational study designs are more susceptible to systematic errors and therefore yield suboptimal evidence for assessing intervention efficacy [13, 14]. Primary biomarkers analyzed included IL-6, TNF-alpha, CRP, and adiponectin. This study considered publications regardless of year or language.

Studies missing either atorvastatin or rosuvastatin were excluded. Additionally, non-randomized controlled trials, cohort studies (prospective or retrospective), case–control studies, cross-sectional studies, case series, animal studies, reviews, editorials, commentaries, meta-analyses, and research articles lacking measurable inflammatory biomarkers were excluded from the analysis.

Two reviewers screened the studies in Covidence using pre-specified inclusion and exclusion criteria. Covidence is a web-based collaboration tool designed to simplify the process of systematic reviews and other types of reviews [15].

Initially, two reviewers independently screened the titles and abstracts of retrieved studies to identify potentially eligible studies. Subsequently, full-text articles of the potentially relevant studies were retrieved and assessed. Two independent co-authors reviewed each study according to our criteria. A third independent reviewer resolved disagreements when consensus was not reached.

Two reviewers independently extracted and cross-checked data for accuracy. A third independent reviewer was consulted to resolve the conflict if consensus was not reached. The following baseline and summary data were collected from the eligible studies, when present: First author’s last name, year of publication, country, study design, sample size, age, gender, BMI, number of smokers, baseline comorbidities, dosage of atorvastatin and rosuvastatin used, baseline medications (antiplatelets and antihypertensives), baseline levels of inflammatory biomarkers (CRP, IL-6, adiponectin, TNF-alpha) and baseline lipid profile levels (total cholesterol, LDL, HDL, Triglycerides). Regarding the outcomes, data on the change in levels of CRP, IL-6, adiponectin, and TNF-alpha following administration of the statin were extracted.

We assessed risk of bias using the Cochrane RoB 2 tool. It provides a framework for considering the risk of bias in the findings of any randomized trial. The evaluation is structured into a series of five domains through which bias might be introduced into a trial: (1) bias arising from the randomization process, (2) bias due to deviations from intended interventions, (3) bias due to missing outcome data, (4) bias in the measurement of the outcome, and (5) bias in the selection of the reported result [16].

We assessed the certainty of evidence for each meta-analysis using the GRADE framework [17]. Evidence certainty was classified as ‘high,’ ‘moderate,’ ‘low,’ or ‘very low.’ As all included studies were randomized controlled trials (RCTs), they were initially rated as ‘high’ certainty. We downgraded certainty by one or more levels: ‘moderate’ (1 level), ‘low’ (2 levels), or ‘very low’ (3 levels), based on factors such as risk of bias, inconsistency, or imprecision. Conversely, the certainty could be upgraded if certain strengthening factors were present, including a large magnitude of effect or a precise dose–response relationship.

This review relied exclusively on secondary data from published studies. Since it does not include human participants, ethical approval was not necessary.

We conducted a double-armed meta-analysis using R software, version 4.4.1 [18]. Our analysis included only continuous outcomes, expressed as mean differences (MDs) with 95% confidence intervals (CIs). For each trial, we extracted sample sizes, means, and standard deviations to calculate the pooled estimates. Before pooling, the units used were as follows: CRP (mg/L), adiponectin (pg/mL), IL-6 (μg/mL), and TNF-alpha (pg/mL).

To account for considerable variation in baseline CRP levels and standard deviations across studies, we also performed a meta-analysis using the standardized mean difference (SMD). This approach provides a scale-independent measure of effect size, allowing for more appropriate comparisons across heterogeneous study populations, particularly when absolute values or measurement scales vary between trials [19].

Because CRP data were often non-normally distributed, means and standard deviations were not always directly available. Instead, they were estimated using the median, range, and sample size [20, 21]. When only baseline and follow-up CRP levels were reported, the standard deviations for changes in CRP concentrations were derived using an imputed correlation coefficient of 0.8, following the guidelines in the Cochrane Handbook [22].

A random-effects model was used for all analyses to account for expected methodological heterogeneity across studies, including differences in populations, interventions, dosages, and follow-up durations. While statistical heterogeneity (e.g., I^2^) was reported for transparency, it was not used as the sole criterion for model selection [23]. To further address heterogeneity, we performed a sensitivity analysis on dosages, follow-up periods, and disease conditions specifically for CRP levels only, as data on other inflammatory biomarkers were scarce. Furthermore, a meta-regression was performed to investigate the sources of heterogeneity in the changes in CRP levels further.

To explore the influence of study quality on the pooled effect, we conducted sensitivity analyses stratified by risk of bias, including separate analyses for studies assessed as having low risk of bias and those with some concerns.

We inspected funnel plots for asymmetry and conducted Egger’s test to assess for publication bias. Results were considered significant if the p-value was less than 0.05 [24].

## Results

Application of our search strategy on Web of Science, Cochrane, PubMed, and Scopus yielded 767 records. After removing duplicates (153 by Covidence and 9 manually), 605 records remained and underwent title and abstract screening. From these, 117 studies were selected for full-text screening.

**Fig. 1:**
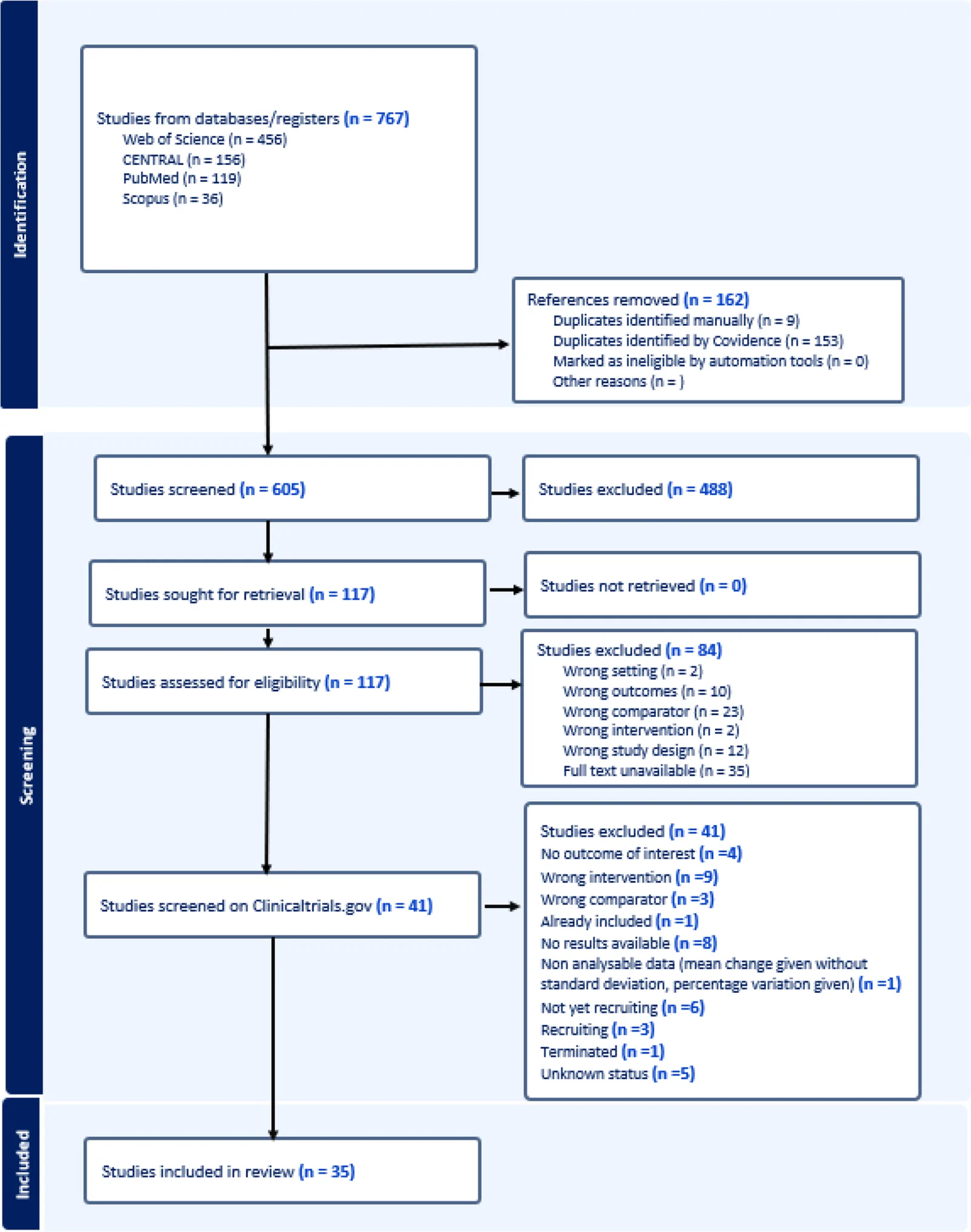
Preferred reporting items for systematic reviews and meta-analyses (PRISMA) flowchart of the screening process

Following the screening process, we identified 33 studies for inclusion. Before conducting the analysis, an additional literature search identified two more studies, bringing the total to 35 [25–59] studies in our systematic review and meta-analysis. Additionally, after completing the review, we searched ClinicalTrials.gov to identify any potentially missing randomized controlled trials (RCTs). This search yielded 41 studies; however, no new eligible studies were added, as the identified trials were either already included, lacked posted results, or were incomplete. Further information is provided in Additional file 1, and the inclusion process is visualized in the PRISMA flowchart Fig. 1.

The studies included diverse populations, with sample sizes ranging from 22 to 1039 participants. The studies were conducted across various countries, including the USA, UK, Netherlands, Greece, Egypt, and several Asian countries. The mean ages of participants ranged from approximately 43 to 69 years. The proportion of male participants varied widely, from 16.7% to 96.8%. Body Mass Index (BMI), reported in several studies, ranged from 23 to 33 kg/m^2^. Smoking prevalence, when noted, varied between 9.1% and 85%. Baseline lipid profiles revealed diverse total cholesterol (166–372 mg/dL), LDL cholesterol (92–292 mg/dL), HDL cholesterol (28–62 mg/dL), and triglycerides (116–363 mg/dL) across studies. Inflammatory biomarkers such as CRP ranged from 1.05 mg/L to 53 mg/L, reflecting varied patient risk levels and comorbidities. Additional information is presented in Table 1 and Additional file 2.

**Table 1:** Baseline demographics and lipid profiles of included participants

Author Year	Country	Intervention	Sample size	Age (Mean, ± SD)(Years)	Male (%)	BMI (Mean ± SD)	Smokers (%)	Baseline lipid profile (Mean ± SD)			
								Total cholesterol (mg/dL)	LDL (mg/dL)	HDL (mg/dL)	Triglyceride (mg/dL)
Shioji 2014 [18]	Japan	ATV	77	69.3 ± 9.5	65	N/A	9.1	N/A	121.4 ± 17.6	57.9 ± 16.7	133.3 ± 73.6
		RSV	75	66.9 ± 11.9	56	N/A	12	N/A	122.9 ± 18.9	57.3 ± 14.6	132.4 ± 66.8
Sindhu 2011 [19]	India	ATV	20	49 ± 8.99	65	32.15 ± 1.4	N/A	243.56 ± 45.15	163.98 ± 25.88	42.73 ± 7.95	173.16 ± 52.0
		RSV	20	49.1 ± 6.82	65	32.1 ± 1.72	N/A	245.93 ± 45.15	173.72 ± 27.80	44.11 ± 8.31	182.07 ± 38.33]
Stein 2003 [20]	USA	ATV	187	47 ± 13	57	28 ± 5	N/A	367 ± 53	288 ± 49	47 ± 12	159 ± 73
		RSV	436	48 ± 14	54	27 ± 5	N/A	372 ± 55	292 ± 51	48 ± 13	160 ± 75
Thongtan 2011 [21]	USA	ATV	135	58.6 ± 11	50	29.4 ± 6.2	N/A	278.3 ± 30.7	197.8 ± 27.2	52.3 ± 14.1	178.1 ± 75.1
		RSV	137	55.9 ± 12.7	52	29.5 ± 12.6	N/A	283.2 ± 26.7	204.4 ± 27.1	50.1 ± 12.6	183.2 ± 70.8
Tian 2018 [22]	China	ATV	30	56.2 ± 8.9	60	N/A	60	228.15 ± 39.06	166.67 ± 59.17	N/A	N/A
		RSV	30	56.8 ± 8.4	60	N/A	63	227.38 ± 46.79	161.25 ± 43.31	N/A	N/A
Tian 2017 [23]	China	ATV	42	43.26 ± 11.78	47.6	24.45 ± 3.82	N/A	248.26 ± 41.76	142.69 ± 30.16	36.74 ± 7.73	151.45 ± 14.17
		RSV	38	46.37 ± 11.43	52.6	23.59 ± 2.15	N/A	256.00 ± 43.70	131.48 ± 25.91	35.58 ± 8.51	155.00 ± 13.29
Tran 2021 [24]	nkVietnam	ATV	48	62.4 ± 12.2	70.8	23.22 ± 2.3	60.4	N/A	133.41 ± 39.06	N/A	N/A
		RSV	48	63.6 ± 11.8	64.6	23.37 ± 2.13	50	N/A	128.38 ± 31.32	N/A	N/A
Umrani 2020 [25]	Pakistan	ATV	54	N/A	N/A	N/A	N/A	N/A	N/A	N/A	N/A
		RSV	59	N/A	N/A	N/A	N/A	N/A	N/A	N/A	N/A
Anagnostis P et al. 2011 [27]	Greece	ATV	18	59.8 ± 10.3	16.7	N/A	N/A	272 ± 41	183 ± 34	58 ± 8	167 ± 90
		RSV	18	54.7 ± 11.3	16.7	N/A	N/A	278 ± 35	187 ± 24	62 ± 17	149 ± 79
Anagnostis et al. 2014 [26]	Greece	ATV	28	56.1 ± 2.2	21.43	30.9 ± 0.8	N/A	284 ± 8	192 ± 6	60 ± 2	166 ± 14
		RSV	24	57.4 ± 1.9	16.67	29.7 ± 0.7	N/A	275 ± 7	185 ± 5	60 ± 3	150 ± 14
Aydin 2015 [28]	Turkey	ATV	59	58 ± 11	76	N/A	46	204 ± 31	144 ± 25	38 ± 8	116 ± 72
		RSV	61	58 ± 11	84	N/A	51	201 ± 35	141 ± 28	38 ± 9	109 ± 67
Brunetti 2007 [29]	Italy	ATV	11	60.2 ± 10	72.7	N/A	27.3	200.3 ± 29.4	99.1 ± 26.1	40.4 ± 4.9	363 ± 184.9
		RSV	11	60.3 ± 7.2	63.6	N/A	18.2	191.6 ± 43.6	105.7 ± 32.3	47.2 ± 13	200.4 ± 128.3
Cao 2017 [30]	China	ATV	60	45.83 ± 7.49	58.33	N/A	11.67	227.38 ± 42.15	145.01 ± 29.78	33.64 ± 8.51	183.34 ± 32.77
		RSV	60	46.75 ± 7.51	50	N/A	13.33	230.86 ± 43.31	150.04 ± 30.55	32.48 ± 8.12	186.88 ± 37.20
Cimato 2015 [31]	USA	ATV	12	43.4 ± 12.5	58.3	24.9 ± 7.2	N/A	210.5 ± 27.6	136.2 ± 22.9	53.5 ± 12.9	N/A
		RSV	12	43.4 ± 12.5	58.3	24.9 ± 7.2	N/A	210.5 ± 27.6	136.2 ± 22.9	53.5 ± 12.9	N/A
Darban 2021 [32]	Iran	ATV	30	60.8 ± 6.5	63.3	N/A	N/A	166.5 ± 33.9	113.2 ± 28.5	42.4 ± 8.2	172.7 ± 63.1
		RSV	30	61.4 ± 6.7	53.3	N/A	N/A	166.4 ± 47.5	111.2 ± 21.1	42.3 ± 6.8	178.6 ± 69.9
Said 2021 [33]	Egypt	ATV	42	44 ± 12.75	73.8	25.39 ± 2.94	38.1	200.24 ± 12.52	130.31 ± 36.99	44.07 ± 15.54	181.4 ± 56.93
		RSV	43	45.77 ± 11.84	81.3	25.78 ± 2.12	48.8	201.82 ± 14.41	129.19 ± 28.37	44.77 ± 16.106	169.2 ± 40.29
Gorji 2023^1^ [34]	Iran	ATV	19	56.6 ± 8.7	47.4	27.64 ± 4	21.1	168.32 ± 48	93.21 ± 26.78	42.6 ± 10.6	139.68 ± 48.86
		RSV	19	53.6 ± 7.2	52.6	26 ± 3.2	31.6	171.65 ± 33.64	94.06 ± 20.76	45.7 ± 8.5	114.61 ± 56.87
Gorji 2023^2^ [34]	Iran	ATV	21	56 ± 9.5	47.6	26 ± 3.18	33.3	163.6 ± 52.47	93.86 ± 28.65	43.1 ± 10	142.94 ± 58.83
		RSV	20	55.2 ± 10.1	45	26.72 ± 4.79	20	165.42 ± 23.14	92.75 ± 25.87	48.5 ± 12.2	116.4 ± 79.78
Hong 2011 [35]	South Korea	ATV	63	58 ± 10	73	N/A	29	181 ± 43	117 ± 38	48 ± 15	124 ± 117
		RSV	65	59 ± 10	75	N/A	37	185 ± 42	122 ± 37	47 ± 10	125 ± 94
Karalis 2010 [36]	Netherlands	ATV	41	64.0 ± 9.8	92.6	28 ± 3.4	N/A	218.92 ± 56.45	143.33 ± 50.50	28.40 ± 2.97	254.85 ± 102.07
		RSV	39	65.8 ± 7.7	94,8	29.0 ± 4.0	N/A	216.80 ± 26.79	140.33 ± 23.81	29.64 ± 5.65	228.86 ± 149.97
Saku 2011 [37]	Japan	ATV	99	61.5 ± 9.5	34.3	23.8 ± 3.4	14.1	N/A	162 ± 25	56.9 ± 13.8	136 ± 68
		RSV	100	61.7 ± 10.3	35	23.9 ± 3.7	12	N/A	171 ± 29	56.6 ± 13.0	140 ± 73
Khurana 2015 [38]	India	ATV	50	N/A	N/A	N/A	N/A	190.92 ± 48.07	101.91 ± 34.47	38.74 ± 10.12	143.66 ± 54.73
		RSV	50	N/A	N/A	N/A	N/A	209.14 ± 48.92	109.13 ± 31.39	40.82 ± 7.52	138.66 ± 43.86
Kumar 2019 [39]	Pakistan	ATV	94	N/A	N/A	N/A	N/A	220.91 ± 35.84	200.49 ± 54.14	39.28 ± 10.54	145.07 ± 48.81
		RSV	99	N/A	N/A	N/A	N/A	223.43 ± 33.56	198.36 ± 53.66	38.78 ± 9.48	143.60 ± 53.39
Milionis 2006 [40]	Greece	ATV	60	53.3 ± 7.7	58.3	24.0 ± 3.4	26.7	285 ± 43	204 ± 40	48 ± 8	157 ± 55
		RSV	60	53.5 ± 7.9	55	24.1 ± 3.2	23.3	285 ± 30	205 ± 42	48 ± 6	159 ± 51
Mostafa 2018 [41]	Egypt	ATV	50	54.6 ± 9	70	N/A	54	191.7 ± 48.1	128 ± 45.3	36.4 ± 9.5	315 ± 437
		RSV	50	54.9 ± 8.4	60	N/A	40	199.2 ± 53.2	139.1 ± 37.6	38.7 ± 13.3	252 ± 307
Namal 2011 [42]	Turkey	ATV	45	62.47 ± 8.40	62.2	N/A	64.4	N/A	141.7 ± 3.69	N/A	N/A
		RSV	46	64.85 ± 11.60	73.9	N/A	56.5	N/A	151.7 ± 3.28	N/A	N/A
Qu 2009 [43]	China	ATV	34	59.2 ± 9.3	47	25.7 ± 1.9	32.3	221.89 ± 41.58	143.26 ± 23.06	39.69 ± 18.14	171.19 ± 100.23
		RSV	35	57.7 ± 11.1	57.1	26.1 ± 1.6	25.7	231.34 ± 34.40	157.25 ± 28.35	45.36 ± 20.79	165.87 ± 83.38
Tsutamoto 2011 [44]	Japan	ATV	32	60.6 ± 10.9	96.8	N/A	N/A	203 ± 34	115 ± 32	42.6 ± 11	190 ± 108
		RSV	31	59.8 ± 8.8	96.77	N/A	N/A	189 ± 34	111 ± 28	43 ± 10	192 ± 80
Bitteridge 2007 [45]	UK	ATV	246	62 ± 11	65	31	N/A	213 ± 41	131 ± 36	46 ± 12	173.69 ± 81.2
		RSV	248	61 ± 11	57	30	N/A	213 ± 38	131 ± 32	46 ± 14	173.269 ± 91.12
Elhadad 2024 [46]	Egypt	ATV	40	53 ± 10	87.5	26 ± 1.5379	85	214 ± 54	136 ± 54	47 ± 12	146.388 ± 74.586
		RSV	40	56 ± 10	80	26.6 ± 2.31	75	202 ± 55	125 ± 58	46 ± 16	157.36 ± 96.11
Nicholls 2011 [47]	USA	ATV	519	57.9 ± 8.5	74.4	29.2 ± 5.5	30.3	193.5 ± 34.2	119.9 ± 28.9	44.7 ± 10.7	134.905 ± 59.471
		RSV	520	57.4 ± 8.6	72.9	28.9 ± 5.0	34.4	193.9 ± 34.1	120.0 ± 27.3	45.3 ± 11.8	133.606 ± 66.9045
Liu B et al. 2011 [48]	China	ATV	18	56.8 ± 10.1	66.7	28.7 ± 4.8	27.8	229.6 ± 31.3	149.7 ± 6.6	56.0 ± 5.2	167.4 ± 35.6
		RSV	18	58.3 ± 9.2	61.1	29.4 ± 4.3	22.2	235.6 ± 30.6	162.3 ± 7.3	50.1 ± 5.3	182.1 ± 25.3
Ferdinand 2006^1^ [49]	USA	ATV	179	54.9 ± 10.3	36.1	32.7 ± 6.8	N/A	269.4 28.1	189.1 ± 24.0	52.5 ± 12.0	139.3 ± 56.4
		RSV	186	55.0 ± 11.2	39.5	32.3 ± 6.2	N/A	270.7 31.9	191.8 ± 27.2	51.5 ± 11.2	136.5 ± 49.7
Ferdinand 2006^2^ [49]	USA	ATV	178	54.9 ± 11.9	33.3	32.4 ± 5.9	N/A	270.8 30.6	191.9 ± 26.6	49.9 ± 12.8	146.3 ± 49.7
		RSV	189	55.4 ± 12.8	32.1	32.5 ± 7.6	N/A	270.9 29.4	189.6 ± 23.4	52.6 ± 14.2	143.7 ± 55.9
Park 2010 [50]	Korea	ATV	178	58.96 ± 0.75	37.64	N/A	N/A	238.55 ± 1.89	163.85 ± 1.62	39.76 ± 0.55	174.75 ± 4.69
		RSV	172	60.49 ± 0.74	42.44	N/A	N/A	237.52 ± 2.07	163.64 ± 1.76	39.66 ± 0.54	171.08 ± 5.20
Puccetti 2011 [51]	Italy	ATV	30	51.8 ± 3.6	60	24.65 ± 1.35	N/A	282.29 ± 7.85	214.43 ± 9.78	49.11 ± 2.66	95.66 ± 9.98
		RSV	30	51.05 ± 2.55	56.67	24.6 ± 1.05	N/A	285 ± 9.33	217.91 ± 11.24	48.53 ± 3.84	93 ± 8.28
Stalenhoef 2005 [52]	Europe & USA	ATV	157	57.3 ± 9.4	67.5	31.1 ± 3.5	N/A	116.46 ± 14.22	78.30 ± 11.70	20.88 ± 4.50	40.50 ± 14.04
		RSV	165	58.1 ± 9.4	60.6	30.5 ± 3.6	N/A	116.64 ± 14.58	79.20 ± 12.60	20.34 ± 4.50	42.12 ± 14.22

Ferdinand 2006^1^ – Refers to a group of patients receiving atorvastatin 10 mg and rosuvastatin 10 mg

Ferdinand 2006^2^ – Refers to a group of patients receiving atorvastatin 20 mg and rosuvastatin 20 mg

Gorji 2023^1^ – Refers to a group of patients receiving atorvastatin 40 mg and rosuvastatin 20 mg

Gorji 2023^2^ – Refers to a group of patients receiving atorvastatin 20 mg and rosuvastatin 10 mg

*ATV* Atorvastatin, *RSV* Rosuvastatin, *LDL* Low-Density Lipoprotein, *HDL* High-Density Lipoprotein, *TG* Triglycerides, *TC* Total Cholesterol

### Risk of bias assessment

**Fig. 2:**
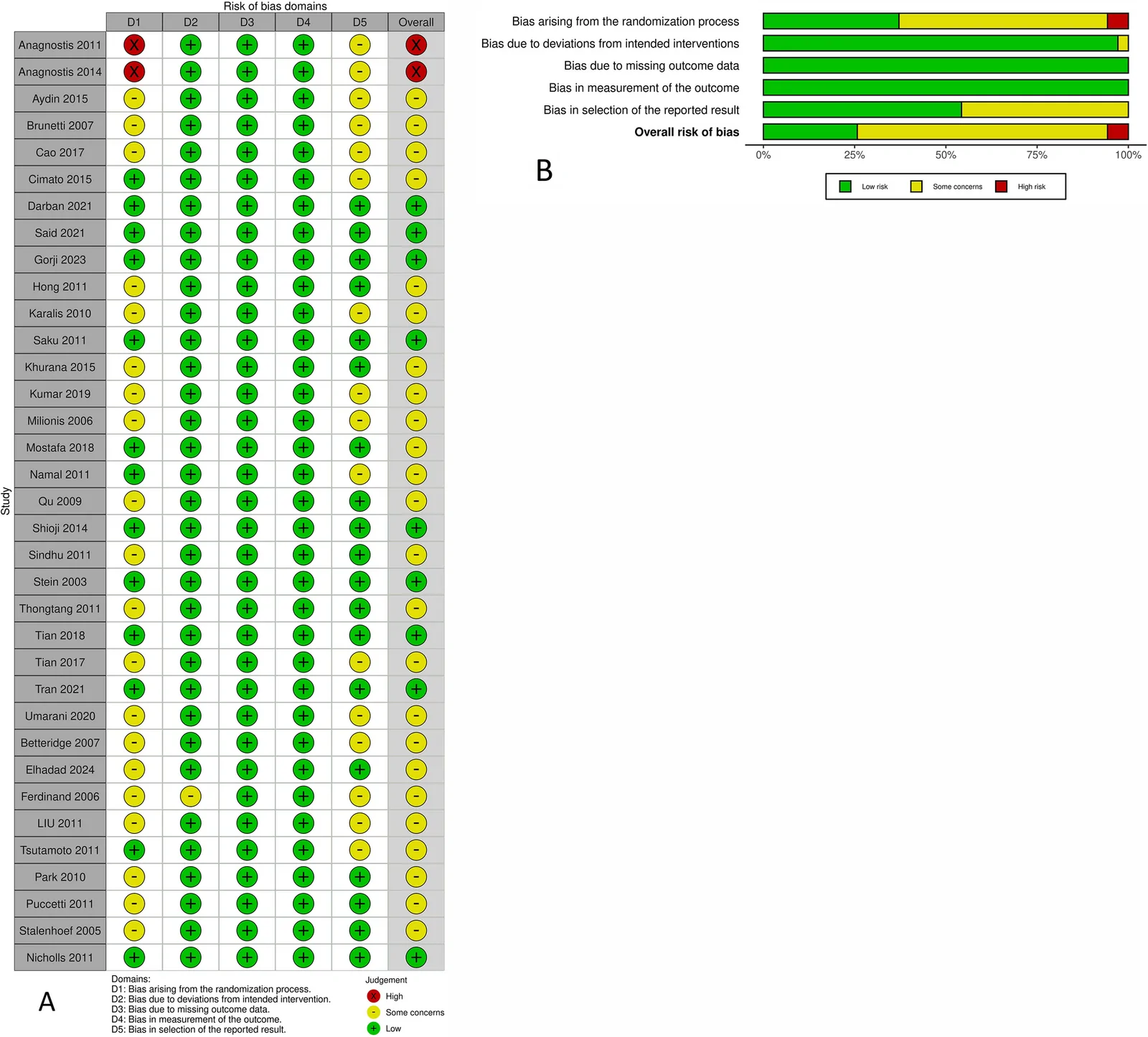
Risk of bias assessment (Traffic light plot (**A**) and summary plot (**B**))

The risk of bias was assessed for all included studies using the Cochrane Risk of Bias 2 (RoB 2) tool. Of the 35 studies evaluated, two were judged to have an overall high risk of bias, 24 studies were classified as having some concerns, and nine studies were determined to have a low risk of bias. Additional results are shown in Fig. 2.

### Decrease in CRP from baseline to follow-up

**Fig. 3:**
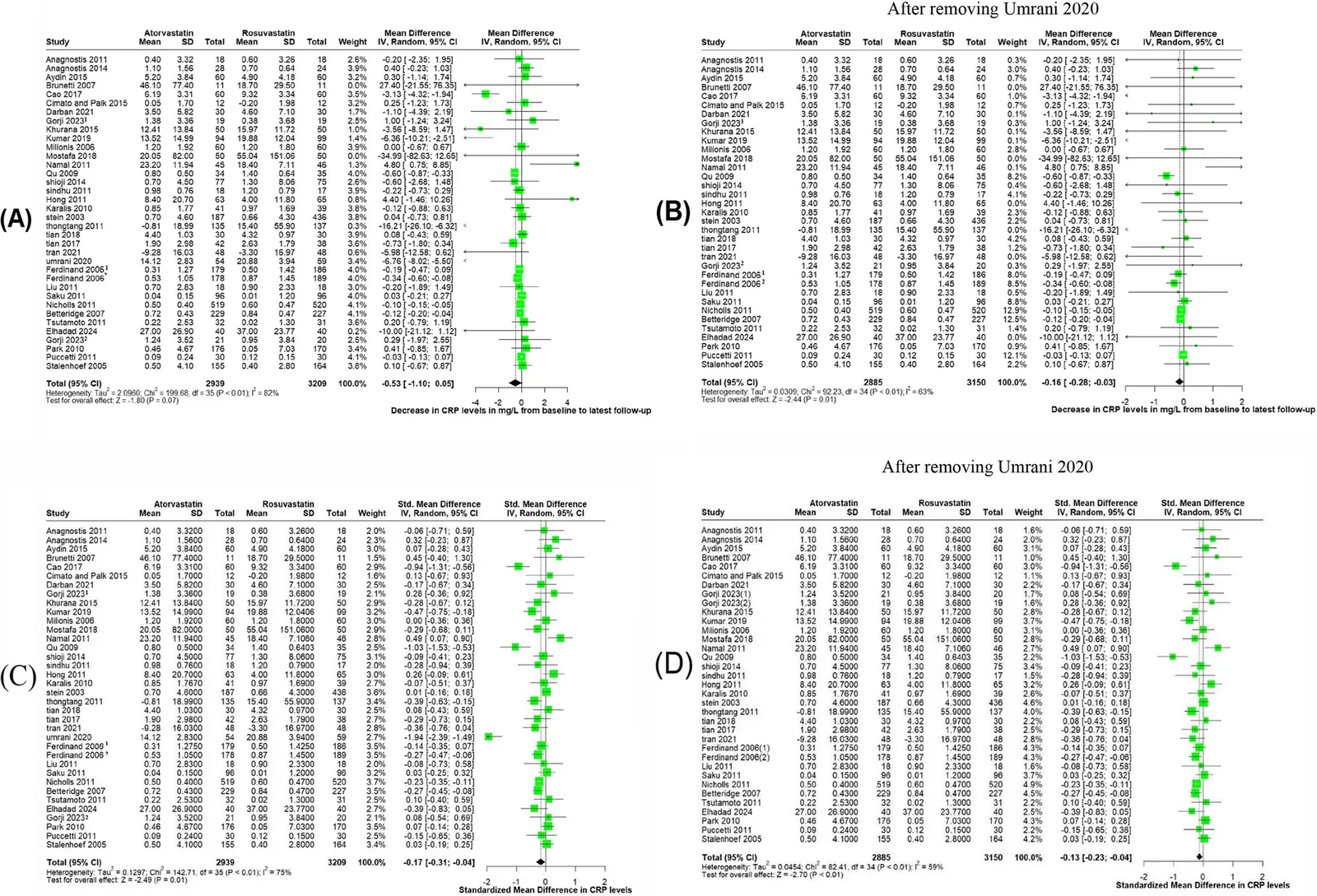
**A** Forest plot depicting the overall decrease in CRP levels in patients from baseline to follow-up (Mean Difference). **B** Forest Plot Depicting the decrease in CRP Levels in Patients from Baseline to Follow-Up (Mean Difference, Post-Sensitivity Analysis by Removing Umrani 2020). **C** Forest plot depicting the overall decrease in CRP levels in patients from baseline to follow-up (Standardized Mean Difference). **D** Forest plot depicting the decrease in CRP levels in patients from baseline to follow-up (Standardized Mean Difference, post-sensitivity analysis excluding Umrani 2020)

We performed a head-to-head meta-analysis of 6,148 participants using a random-effects model. The follow-up period was defined as the latest available time point reported in each study. There was no statistically significant difference between atorvastatin (*n* = 2,939) and rosuvastatin (*n* = 3,209) (MD [95% CI]: − 0.53 [− 1.10 to 0.05], *p* = 0.07, I^2^ = 82%, certainty of evidence: low) (Fig. 3A, Table 2). To address the high heterogeneity, we performed a leave-one-out test. Excluding Umrani et al. reduced heterogeneity and yielded a greater CRP reduction with rosuvastatin (MD [95% CI]: − 0.16 [− 0.28 to − 0.03], *p* = 0.01, I^2^ = 63%, certainty of evidence: moderate) (Fig. 3B, Table 2).

**Table 2:** GRADE summary of the certainty of evidence for CRP and other inflammatory biomarkers

Outcome	No. of Studies	Risk of Bias	Inconsistency	Indirectness	Imprecision	Publication Bias	Effect Size	Quality of Evidence
CRP (MD – Before Sensitivity)	34	Some concerns	High (I^2^ = 82%)	No	High (CI: − 1.10 to 0.05)	None (Egger’s p = 0.09)	MD = − 0.53 (− 1.10 to 0.05), p = 0.07	Low
CRP (MD – After removing Umrani et al.)	33	Some concerns	Moderate (I^2^ = 63%)	No	Low (CI: − 0.28 to − 0.03)	Not assessed	MD = − 0.16 (− 0.28 to − 0.03), p = 0.01	Moderate
CRP (MD – Low RoB)	9	Low	None (I^2^ = 0%)	No	Low (CI: − 0.16 to − 0.01)	Not assessed	MD = − 0.08 (− 0.16 to − 0.01), p = 0.03	Moderate
CRP (MD – Some Concerns RoB)	24	Some concerns	High (I^2^ = 87%)	No	High (CI: − 1.70 to 0.10)	Not assessed	MD = − 0.80 (− 1.70 to 0.10), p = 0.08	Low
CRP (SMD – Before Sensitivity)	34	Some concerns	High (I^2^ = 75%)	No	Low (CI: − 0.31 to − 0.04)	None (Egger’s p = 0.09)	SMD = − 0.17 (− 0.31 to − 0.04), p = 0.01	Moderate
CRP (SMD – After removing Umrani et al.)	33	Some concerns	Moderate (I^2^ = 59%)	No	Low (CI: − 0.23 to − 0.04)	Not assessed	SMD = − 0.13 (− 0.23 to − 0.04), p < 0.01	Moderate
CRP (SMD – Low RoB)	9	Low	Low (I^2^ = 22%)	No	High (CI: − 0.21 to 0.03)	Not assessed	SMD = − 0.09 (− 0.21 to 0.03), p = 0.14	Low
CRP (SMD – Some Concerns RoB)	24	Some concerns	High (I^2^ = 81%)	No	Low (CI: − 0.42 to − 0.05)	Not assessed	SMD = − 0.24 (− 0.42 to − 0.05), p = 0.01	Moderate
IL-6	7	Some concerns	Moderate (I^2^ = 65%)	No	High (CI: − 3.02 to 1.38)	Not assessed	MD = − 0.82 (− 3.02 to 1.38), p = 0.46	Low
Adiponectin	4	High	Low (I^2^ = 7%)	No	High (CI: − 4.70 to 1.14)	Not assessed	MD = − 1.78 (− 4.70 to 1.14), p = 0.23	Low
TNF-α	4	Some concerns	Moderate (I^2^ = 66%)	No	High (CI: − 0.05 to 0.52)	Not assessed	MD = 0.23 (− 0.05 to 0.52), p = 0.11	Low

*MD* Mean Difference, *SMD* Standardized Mean Difference, *CI* Confidence Interval

To test robustness by study quality, we ran a sensitivity analysis restricted to low-risk-of-bias trials. Among nine trials (atorvastatin *n* = 1,027; rosuvastatin *n* = 1,274), rosuvastatin produced a modest but significant additional reduction (MD [95% CI]: − 0.08 [− 0.16 to − 0.01], *p* = 0.03, I^2^ = 0%) (Additional file 3[A], Table 2). I^2^ = 0% supports the robustness of this estimate. In a parallel sensitivity analysis of 25 trials rated as “some concerns” (atorvastatin *n* = 1,866; rosuvastatin *n* = 1,893), the effect was not significant (MD [95% CI]: − 0.80 [− 1.70 to 0.10], *p* = 0.08), with substantial heterogeneity (I^2^ = 87%) (Additional file 3[B], Table 2).

Several individual trials (e.g., Cao 2017, Kumar 2012, Thongtang 2013, Nicholls 2011, Betteridge 2007) favored rosuvastatin, whereas one study (Namal 2018) favored atorvastatin.

Because baseline CRP and SDs varied across studies, we also pooled standardized mean differences (SMDs). The pooled SMD favored rosuvastatin (SMD [95% CI]: − 0.17 [− 0.31 to − 0.04], I^2^ = 75%, *p* = 0.01, certainty of evidence: moderate) (Fig. 3C, Table 2). Excluding Umrani et al. retained significance and lowered heterogeneity (SMD [95% CI]: − 0.13 [− 0.23 to − 0.04], I^2^ = 59%, *p* < 0.01, certainty of evidence: moderate) (Fig. 3D, Table 2).

In low-risk trials (*n* = 9), the SMD was small and non-significant (− 0.09 (95% CI: − 0.21 to 0.03, *p* = 0.14; I^2^ = 22%) (Additional file 3[C], Table 2). In contrast, among the 25 studies with some concerns, the SMD favored rosuvastatin (SMD [95% CI]: − 0.24 [− 0.42 to − 0.05], *p* = 0.01; I^2^ = 81%) (Additional file 3[D], Table 2).

We also analyzed subgroups by condition, follow-up, and dose.

**Fig. 4:**
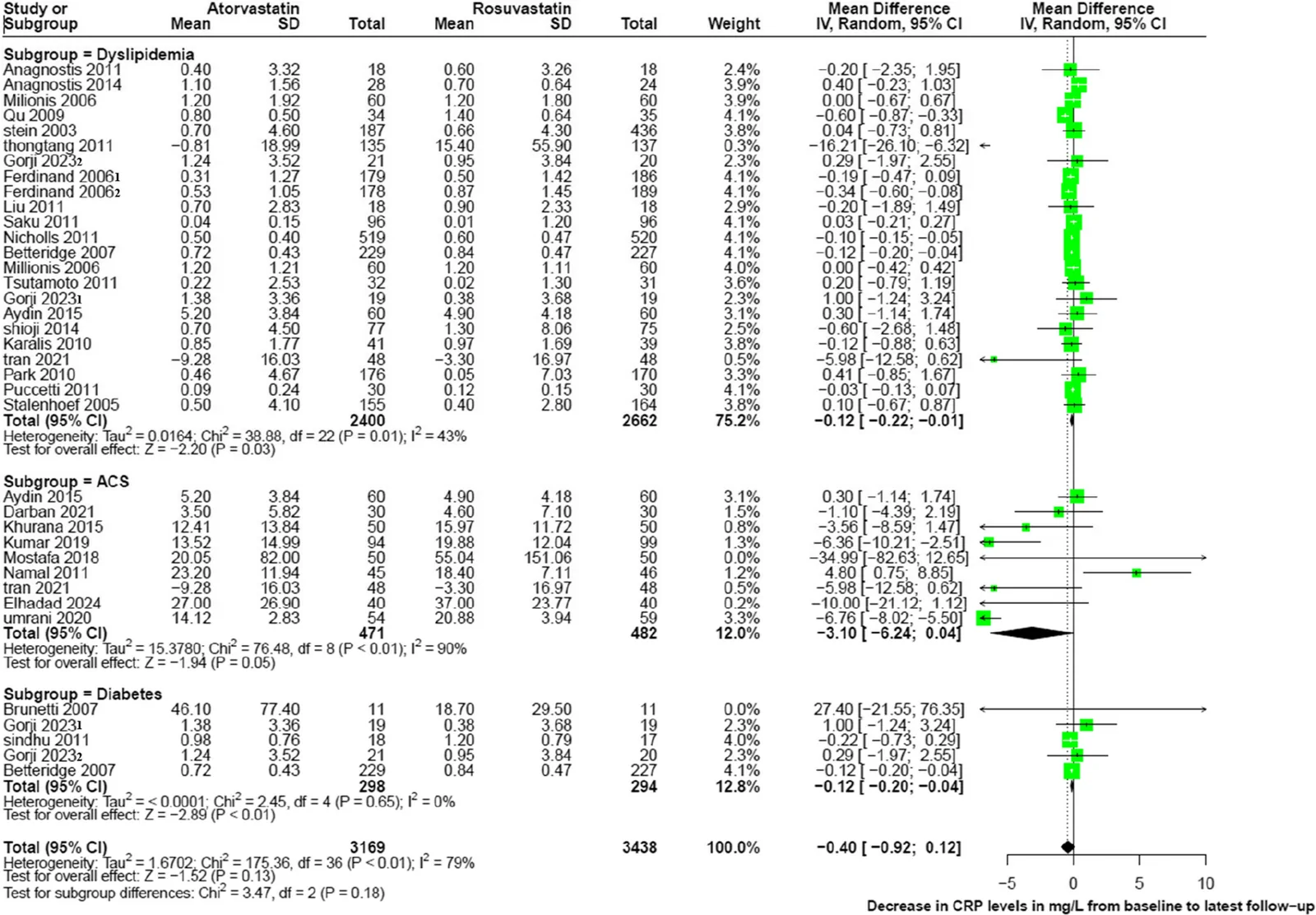
Forest plot depicting the decrease in CRP Levels from baseline to follow-up by disease subgroup

In ACS, rosuvastatin showed a non-significant trend toward benefit compared with atorvastatin (MD [95% CI]: −3.10 [−6.24 to 0.04], I^2^ = 90%, *p* = 0.05). In the dyslipidemia and diabetes subgroups, rosuvastatin was favored (I^2^ = 47%, *p* = 0.03, and I^2^ = 0%, *p* < 0.01, respectively) (Fig. 4).

**Fig. 5:**
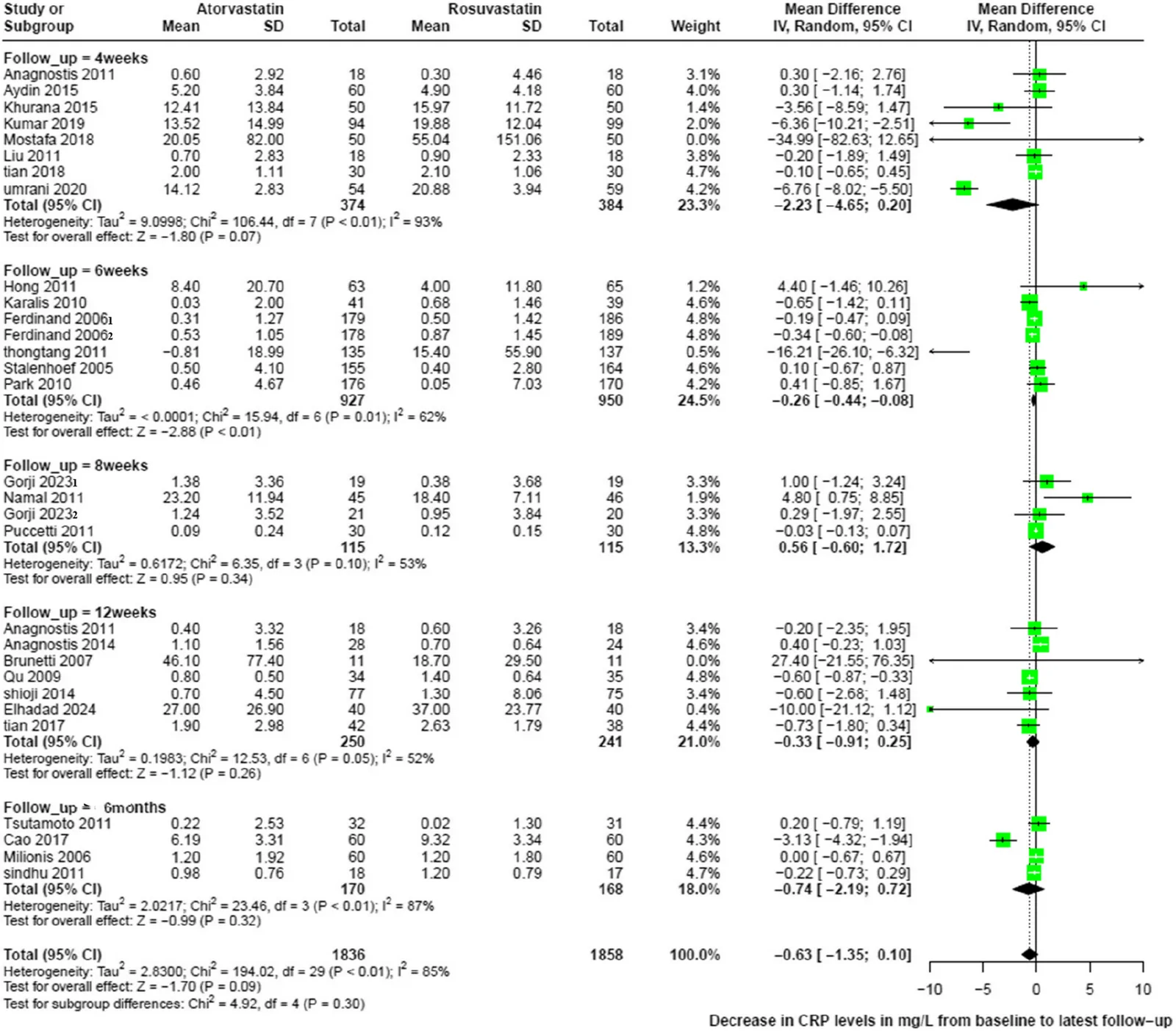
Forest plot depicting the decrease in CRP levels from baseline to follow-up by follow-up period subgroup

The follow-up period subgroup analysis, which included durations of 4 weeks, 8 weeks, 12 weeks, and ≥ 6 months, showed no significant differences between the atorvastatin and rosuvastatin groups. However, at 6 weeks, rosuvastatin showed a greater reduction (MD [95%CI] = − 0.26 [− 0.44 to − 0.08], *p* < 0.01) (Fig. 5).

**Fig. 6:**
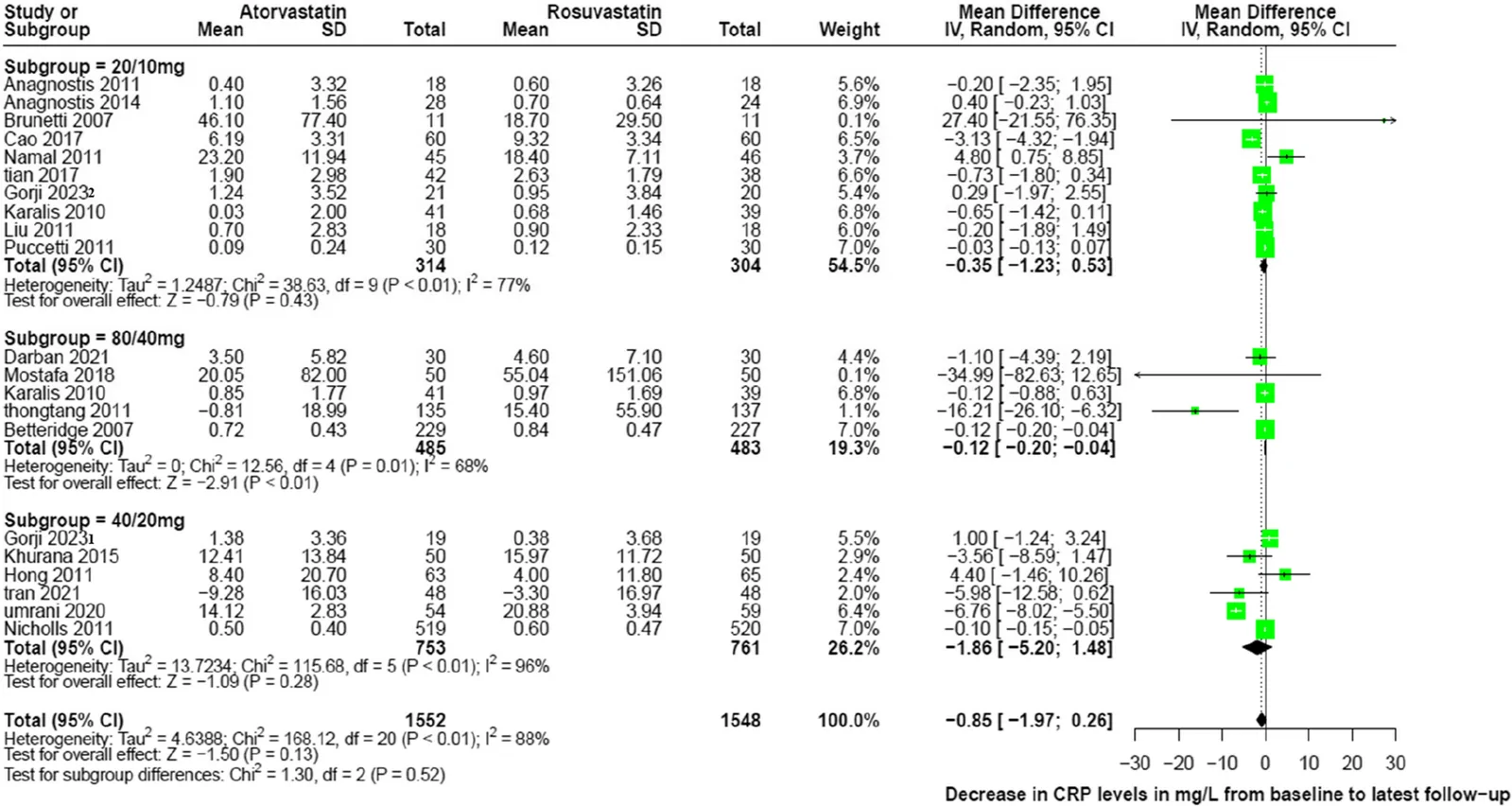
Forest plot depicting the decrease in CRP levels from baseline to follow-up by dosage subgroup. Subgroup analysis was performed according to drug dosages (atorvastatin/rosuvastatin: 20/10 mg, 80/40 mg, and 40/20 mg)

The dose subgroup showed no statistically significant differences between the atorvastatin/rosuvastatin 20/10 mg (*p* = 0.43) and 40/20 mg (*p* = 0.28) dosages. However, at the 80/40 mg dosage, rosuvastatin achieved greater CRP reduction (*p* < 0.01) (Fig. 6).

We conducted a univariate meta-regression analysis using nine covariates to explore potential sources of heterogeneity in CRP changes across studies. These included: mean age, baseline CRP, LDL, HDL, triglycerides, total cholesterol, BMI, percentage of hypertensive patients, and percentage of male participants (Table 3, Additional file 4).

**Table 3:** Univariate meta-regression

	SE	Z-value	Estimate	95% CI	*P*-value	R^2^
Age (Mean)	0.42	−1.53	−0.65	−1.48 to 0.18	0.13	0.00
Baseline CRP (Mean)	0.07	−2.26	−0.16	−0.29 to −0.02	0.02*	0.00
Baseline LDL (Mean)	0.31	−0.96	−0.30	−0.91 to 0.31	0.34	0.00
Baseline HDL (Mean)	0.50	−2.86	−1.44	−2.43 to −0.46	< 0.001*	0.25
Baseline TG (Mean)	0.32	0.90	0.29	−0.34 to 0.91	0.37	0.14
Baseline TC (Mean)	0.44	−0.80	−0.35	−1.22 to 0.51	0.42	0.00
Baseline BMI (Mean)	0.22	1.63	0.36	−0.08 to 0.80	0.10	0.00
Hypertension (%)	0.30	−3.82	−1.16	−1.75 to −0.56	< 0.001*	0.94
Male (%)	0.23	−0.78	−0.18	−0.63 to 0.27	0.44	0.00

*LDL* Low-Density Lipoprotein, *HDL* High-Density Lipoprotein, *TG* Triglycerides, *TC* Total Cholesterol, *CRP* C-Reactive Protein, *CI* Confidence Interval, *SE* Standard Error

^*^Indicates statistical significance (*p* < 0.05)

Three variables showed a negative association with CRP change:

- Baseline CRP (*p* = 0.02),
- Baseline HDL (*p* < 0.001),
- Percentage of hypertensive patients (*p* < 0.001).

These findings suggest that patients with higher baseline CRP, higher HDL, and a greater prevalence of hypertension experienced larger reductions in CRP levels following statin therapy. Baseline CRP reached statistical significance but explained no between-study variance (R^2^ = 0.00). By contrast, hypertension prevalence explained 94% (R^2^ = 0.94) and baseline HDL 25% (R^2^ = 0.25). These findings suggest that hypertension prevalence is a major driver of between-study variability, whereas baseline CRP may act as a within-study effect modifier.

### Decrease in IL-6 levels from baseline to follow-up

**Fig. 7:**
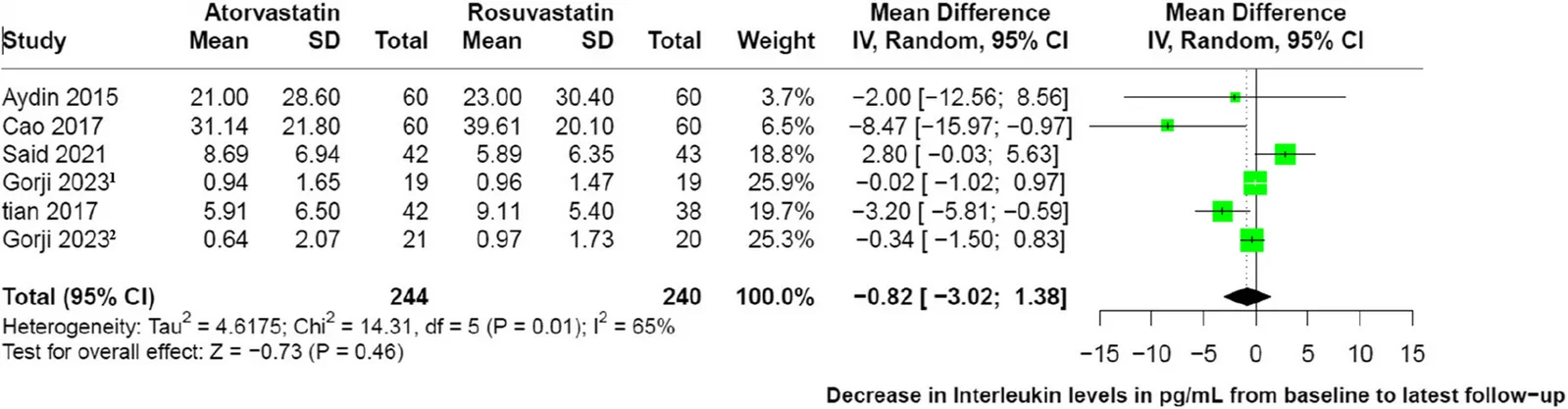
Forest plot depicting the decrease in IL-6 levels from baseline to follow-up

Our analysis of IL-6 levels over the follow-up period, using a random-effects model, revealed no statistically significant difference between the 244 individuals taking atorvastatin and the 240 individuals on rosuvastatin (MD = − 0.82, 95% CI: − 3.02 to 1.38, *p* = 0.46, certainty of evidence: low). The high heterogeneity (I^2^ = 65%) observed suggests variability across studies. Only two studies, by Cao et al. and Tian et al., showed a statistically significant preference for rosuvastatin in reducing IL-6 levels. The remaining studies showed no significant difference (Fig. 7, Table 2).

### Decrease in adiponectin levels from baseline to follow-up

**Fig. 8:**
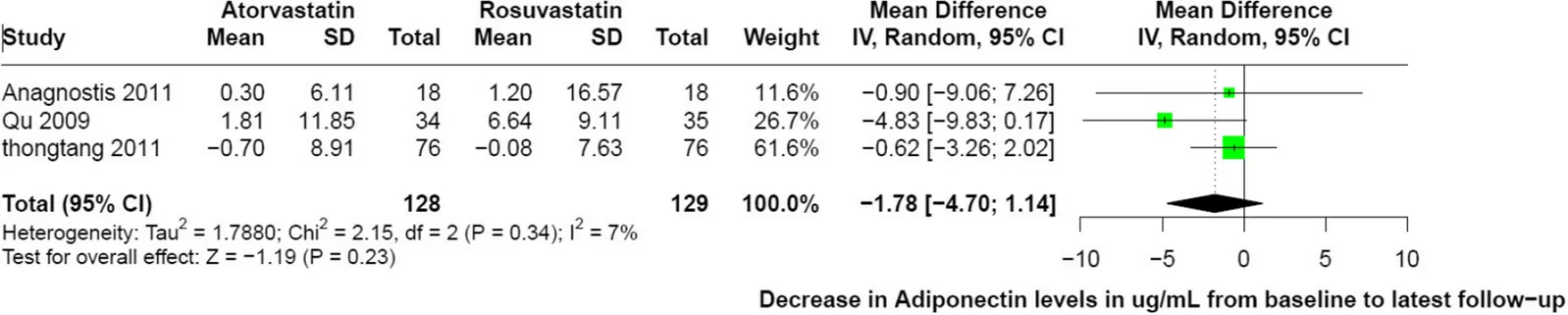
Forest plot depicting the decrease in adiponectin levels from baseline to follow-up

For adiponectin, pooled data from 257 participants indicated no statistically significant difference between the 128 individuals taking atorvastatin and the 129 individuals on rosuvastatin during follow-up (MD = − 1.78, 95% CI: − 4.70 to 1.14, *p* = 0.23, certainty of evidence: low). The heterogeneity was low (I^2^ = 7%), indicating minimal variability across studies. No study demonstrated a significant preference for either drug (Fig. 8, Table 2).

### Decrease in TNF-alpha levels from baseline to follow-up

**Fig. 9:**
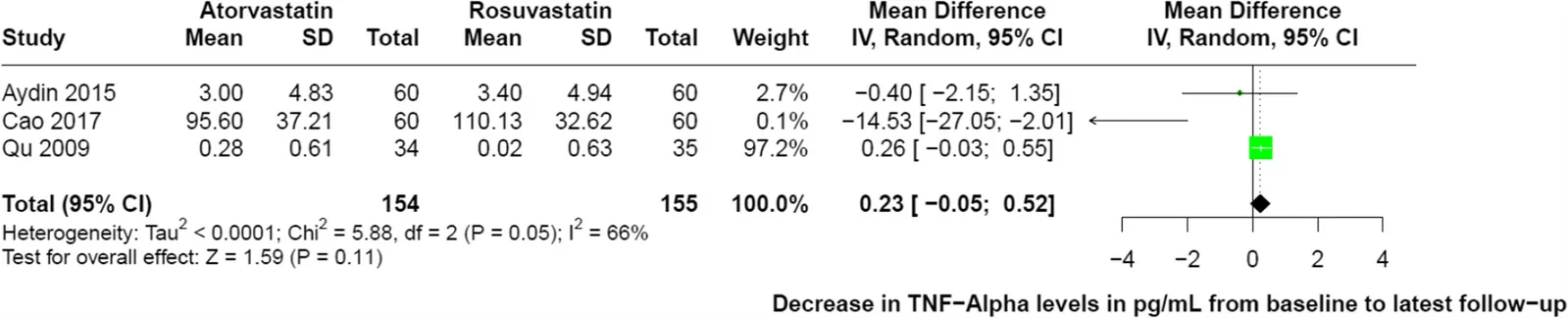
Forest plot depicting the decrease in TNF-alpha levels from baseline to follow-up

Our pooled analysis of 309 individuals showed no statistically significant difference in TNF-alpha levels between the atorvastatin group (*n* = 154) and the rosuvastatin group (*n* = 155) (MD = 0.23, 95% CI: −0.05 to 0.52, *p* = 0.11, certainty of evidence: low) (Fig. 9, Table 2).

The heterogeneity was moderately high (I^2^ = 66%), suggesting variability across studies. Among individual trials, Cao et al. reported a significant reduction in TNF-alpha favoring rosuvastatin (MD = −14.53, 95% CI: −27.05 to −2.01), whereas the remaining studies did not demonstrate significant differences.

### Publication bias

Funnel plots were generated to evaluate potential publication bias. For mean difference outcomes, visual inspection suggested slight asymmetry, driven by a few extreme outliers. However, Egger’s test did not reach statistical significance (*p* = 0.09), indicating no evidence of publication bias. For standardized mean difference outcomes, the contour-enhanced funnel plot appeared more symmetric, with most studies clustered in the nonsignificant region (white zone). Only a few scattered studies lie within the conventionally significant contours (grey/dark grey zones). This pattern suggests that the asymmetry observed in the mean difference analysis was more likely attributable to heterogeneity in baseline CRP values rather than selective publication of positive findings (Additional file 5).

## Discussion

Our comparative study examined how atorvastatin and rosuvastatin affected inflammatory biomarkers, revealing that rosuvastatin was more effective in lowering CRP levels, particularly in sensitivity analyses. In subgroup analyses, rosuvastatin was superior in patients with diabetes or dyslipidemia and at higher doses (80/40 mg). It also showed short-term benefits at six weeks. The meta-regression analysis showed that higher baseline CRP, elevated HDL, and a greater proportion of hypertensive individuals were associated with more pronounced CRP reductions. In contrast, no significant differences were observed for IL-6, adiponectin, or TNF-alpha.

While all studies reported CRP in the same unit (mg/L), differences in baseline CRP levels and variation in measurement precision across populations posed a challenge for direct comparison. The use of SMD helped normalize these differences, yielding a standardized effect size. Although small (SMD = − 0.17), the effect remained statistically significant, supporting rosuvastatin’s modest anti-inflammatory advantage across populations.

Previously published meta-analyses reported contrasting findings on CRP reduction. Takagi et al. (14 RCTs, *n* = 2,696) found no significant difference between atorvastatin and rosuvastatin, whereas our study, with a larger sample size (35 RCTs, *n* = 6,148), favored rosuvastatin (MD [95% CI]: − 0.16 [− 0.28 to − 0.03], *p* = 0.01). Similarly, Ma et al. (13 RCTs, *n* = 3,798) analyzed only dyslipidemia patients and found a modest benefit of rosuvastatin (MD [95% CI]: − 0.12 [− 0.22 to − 0.01], *p* = 0.03), consistent with our subgroup results [10, 11]. Compared with these smaller, more limited analyses, our broader dataset and inclusion of additional biomarkers strengthen the robustness and generalizability of our findings.

Recent literature has expanded our understanding of CRP, showing its involvement in both inflammation and atherosclerosis progression [60]. Other inflammatory biomarkers play indirect roles with CRP, contributing to systemic inflammation characterized by endothelial dysfunction, lipid penetration into the subendothelial space, and fatty streak formation involving foam cells, T-lymphocytes, platelets, and smooth muscle cells. T-lymphocytes within the intima differentiate into Th1 effector cells, which produce cytokines such as IL-1, IFN-γ, IL-6, and TNF-alpha. This is accompanied by an increase in serum inflammatory biomarkers, including CRP, IL-6, and TNF-alpha [61, 62]. Consequently, atherosclerosis progresses through plaque formation and increased blood viscosity, ultimately leading to intimal thickening and plaque rupture [37]. These findings highlight the importance of controlling inflammatory processes alongside lipid management. However, a meta-regression of randomized studies found that while CRP reduction was associated with a lower risk of myocardial infarction, it did not correlate with reduced risk of stroke or all-cause mortality. This suggests that CRP levels may not be a reliable predictor of statin effectiveness [63]. In contrast, Ridker et al. reported that patients with lower CRP levels after statin therapy had better outcomes regardless of LDL levels, supporting additional benefits of statins beyond lipid lowering [64]. Similarly, a large prospective cohort study by Burger et al. showed that elevated CRP levels were independently associated with higher risks of recurrent cardiovascular events and all-cause mortality in patients with CAD, stroke, PAD, and AAA. These associations persisted over long-term follow-up, underscoring the prognostic relevance of CRP across multiple vascular conditions [65].

Statins provide multiple benefits, including lipid-lowering effects, anti-inflammatory actions, improvement in endothelial function, and attenuation of cardiac remodeling [66]. The proposed mechanisms by which statins modulate inflammatory biomarkers include inhibition of lymphocyte adhesion to intercellular adhesion molecule-1 (ICAM-1), thereby preventing T-cell stimulation. Studies also show that statins reduce CRP and IL-6 in human hepatocytes by inhibiting protein prenylation [45, 67, 68]. The greater CRP reduction observed with rosuvastatin compared to atorvastatin may be explained by its longer half-life and lower first-pass hepatic metabolism. Due to its hydrophilic structure, rosuvastatin undergoes less metabolism by cytochrome P450 (CYP) enzymes, in contrast to lipophilic statins such as atorvastatin [69].

The anti-inflammatory effects of statins are thought to improve endothelial function, potentially translating into long-term cardiovascular benefits [70]. However, the evidence in specific patient groups is inconsistent. The impact of statins on endothelial function, typically assessed by flow-mediated vasodilation (FMD) and the vasodilator response to intrabrachial acetylcholine (ACh) using venous occlusion plethysmography, remains controversial in familial hyperlipidemia. For example, one randomized controlled trial found that rosuvastatin did not improve endothelial function after 12 weeks, despite lowering CRP [71]. Conversely, Link et al. reported that rosuvastatin significantly reduced inflammatory biomarkers, including TNF-alpha, a contributor to endothelial dysfunction, in patients with acute coronary syndrome (ACS) [72, 73].

A subgroup analysis from Zhang et al.’s network meta-analysis (NMA) concluded that atorvastatin 80 mg achieved the greatest long-term (> 12 months) CRP reduction, while simvastatin 40 mg was most effective in the short term. These results differ from our findings, which favored rosuvastatin in the short term (6 weeks; *p* ≤ 0.01) but found no difference in the long term (> 6 months; *p* = 0.32). This discrepancy likely arises because most NMA studies compared simvastatin or atorvastatin with placebo, whereas rosuvastatin was assessed across multiple dosages, introducing potential bias. Moreover, the absence of a direct comparison between atorvastatin and rosuvastatin in the NMA limits its ability to determine which statin may be most effective in reducing inflammation [74].

While no universally accepted minimal clinically important difference (MCID) for CRP has been defined in the context of statin therapy, multiple observational studies suggest that even modest changes carry prognostic value. Burger et al. reported that each 1 mg/L increase in CRP was independently associated with an 8% higher risk of recurrent cardiovascular events (HR 1.08; 95% CI: 1.05–1.10) among patients with coronary, cerebrovascular, and peripheral artery disease [65]. Similarly, a large meta-analysis in individuals without established cardiovascular disease found that a threefold increase in CRP, equivalent to a one standard deviation rise in log-transformed levels, was associated with a 27–55% greater risk of major adverse cardiovascular events. The median baseline CRP in this cohort was 1.72 mg/L, indicating that even moderate elevations may be clinically meaningful [75]. Moreover, in patients with coronary artery disease, CRP > 3 mg/L has consistently predicted worse outcomes, with a 37–62% higher risk of recurrent events and a 57–245% higher risk of all-cause mortality compared with CRP < 1 mg/L [65]. Together, these findings underscore the clinical importance of relatively small changes in CRP [65].

The clinical relevance of these biomarker differences between rosuvastatin and atorvastatin remains uncertain. Despite rosuvastatin achieving greater CRP reduction than atorvastatin in our analysis, randomized outcome trials have not demonstrated parallel improvements in hard cardiovascular endpoints. For instance, Nicholls et al. (*n* = 1,578, 104-week follow-up) found no difference between the two statins in major adverse cardiovascular events (MACE), percent atheroma volume, or total atheroma volume, despite greater CRP lowering with rosuvastatin [54]. Likewise, a meta-analysis by Singh et al. (8 RCTs, > 8,700 patients) reported no significant between-statin differences in MACE, all-cause mortality, myocardial infarction, stroke, or revascularization [76]. The ROMA II trial also showed similar outcomes between the drugs, with both outperforming control at all follow-up points, with no between-statin difference in post-procedural myocardial necrosis or MACCE [77]. These results suggest that while CRP reduction has prognostic relevance, biomarker changes alone may not translate into superior long-term clinical benefit when directly comparing rosuvastatin and atorvastatin.

Beyond CRP, our study also provides the first pooled analysis directly comparing non-CRP inflammatory biomarkers between rosuvastatin and atorvastatin. Cao et al. and Tian et al. reported reduced IL-6 levels with rosuvastatin, while other studies found no difference between the drugs [30, 35, 37, 40, 41]. The reasons for these discrepant findings remain unclear. Future studies with larger and more diverse populations are needed to clarify whether one drug is superior.

Cao et al. reported greater TNF-alpha reduction with rosuvastatin, possibly because their cohort had markedly elevated baseline TNF-alpha levels [30, 37]. For adiponectin, no study has demonstrated a difference between the drugs [28, 33, 50]. However, recent evidence suggests a reciprocal relationship between adiponectin and CRP levels. Ouchi et al. found lower adiponectin and higher hs-CRP levels in CAD patients compared with controls. This imbalance may contribute to atherosclerosis, but further studies are required to confirm this [78].

### Strengths

We present a comprehensive analysis of the comparative effectiveness of atorvastatin and rosuvastatin in reducing inflammatory biomarkers, conducted without language or time-of-publication restrictions. To our knowledge, this is the first pooled analysis to evaluate inflammatory biomarkers beyond CRP. By including only randomized controlled trials (RCTs), the study provides high-quality evidence with minimized risk of bias.

Subgroup analyses further reinforced the findings, consistently supporting rosuvastatin’s superiority over atorvastatin in reducing CRP levels across various diseases, follow-up durations, and dosages. In addition, we performed meta-regression on nine clinical and biochemical variables, including age, baseline CRP, lipid profile, BMI, hypertension, and sex, which identified key factors influencing CRP reduction and strengthened the validity of our results.

### Limitations

Several limitations should be noted. Some studies were open-label rather than blinded, which could have introduced bias. The overall duration of follow-up was short, with all trials limited to six months or less. Sample sizes were also small for non-CRP biomarkers, and substantial heterogeneity was observed across studies.

Some trials included only hospitalized patients, which may reduce the generalizability of the findings. In addition, most studies did not perform serial follow-up of serum hs-CRP levels, limiting the ability to evaluate how changes in CRP influenced plaque progression or regression.

Variation in baseline CRP levels, particularly among more severely ill patients, was initially considered a possible contributor to heterogeneity. However, meta-regression showed that although baseline CRP was statistically significant, it explained none of the between-study variance (R^2^ = 0.0).

In cases where standard deviations were not available, we imputed them using methods from the Cochrane Handbook [22]. While necessary, this introduces some uncertainty in effect estimates.

Finally, the exclusion of grey literature and the lack of additional trial registry searches beyond ClinicalTrials.gov (e.g., WHO ICTRP) may have reduced the completeness of the evidence base and contributed to publication bias.

## Conclusion

This meta-analysis, synthesizing evidence from 35 randomized controlled trials with over 6,000 participants, shows that rosuvastatin provides a modest but statistically significant advantage over atorvastatin in reducing CRP levels, particularly at higher doses, in patients with diabetes or dyslipidemia, and during short-term follow-up.

For other inflammatory biomarkers, including IL-6, adiponectin, and TNF-alpha, the evidence remains inconclusive, with no consistent differences observed between the two statins. Sensitivity and meta-regression analyses identified patient characteristics such as baseline CRP, HDL levels, and hypertension prevalence as important modifiers of CRP response, suggesting that patient profiles may influence treatment benefit.

Although rosuvastatin’s greater reduction in CRP supports its stronger anti-inflammatory profile, current evidence does not demonstrate corresponding improvements in long-term cardiovascular outcomes compared with atorvastatin. Therefore, treatment decisions should continue to weigh lipid-lowering efficacy, tolerability, comorbidities, and overall risk–benefit considerations.

Our findings provide the most comprehensive synthesis to date of head-to-head evidence on the anti-inflammatory effects of atorvastatin and rosuvastatin. Future large-scale, long-term RCTs are needed to clarify whether the biomarker differences translate into meaningful clinical outcome advantages.

## Supplementary Information

Additional file 1: Detailed search strategy for each of the included databases.

Additional file 2: Baseline medications, inflammatory biomarkers, and comorbidities of included participants.

Additional file 3: (A) Forest plot depicting the decrease in CRP levels from baseline to follow-up in studies judged to have low risk of bias (Mean Difference). (B) Forest plot depicting the decrease in CRP levels from baseline to follow-up in studies with some concerns for risk of bias (Mean Difference). (C) Forest plot depicting the decrease in CRP levels from baseline to follow-up in studies judged to have low risk of bias (Standardized Mean Difference). (D) Forest plot depicting the decrease in CRP levels from baseline to follow-up in studies with some concerns for risk of bias (Standardized Mean Difference).

Additional file 4: Univariate meta-regression analysis: Mean age [A], baseline CRP [B], baseline LDL [C], baseline HDL [D], baseline triglycerides [E], baseline total cholesterol [F], baseline BMI [G], percentage of hypertensive patients [H], and percentage of male participants [I].

Additional file 5: Funnel plots to assess the publication bias in CRP reduction (A: mean difference, B: standardized mean difference).

Additional file 6: PRISMA 2020 abstract checklist.

Additional file 7: PRISMA 2020 checklist.

## Data Availability

Data is available upon request to the corresponding author.

## Abbreviations

ACS: Acute Coronary Syndrome
Ach: Acetylcholine
AAA: Abdominal Aortic Aneurysm
BMI: Body Mass Index
CAD: Coronary Artery Disease
Cis: Confidence Intervals
CRP: C-Reactive Protein
CVD: Cardiovascular Disease
CeVD: Cerebrovascular Disease
DALYs: Disability-Adjusted Life Years
FMD: Flow-Mediated Dilation
GRADE: Grading of Recommendations Assessment, Development and Evaluation
HDL: High-Density Lipoprotein
HMG-CoA: 3-Hydroxy-3-Methylglutaryl Coenzyme A
IFN-γ: Interferon Gamma
IL-6: Interleukin 6
I^2^: I-squared (measure of heterogeneity)
LDL: Low-Density Lipoprotein
MACE: Major Adverse Cardiovascular Events
MCID: Minimal Clinically Important Difference
MD: Mean Difference
NMA: Network Meta-Analysis
PAD: Peripheral Artery Disease
PRISMA: Preferred Reporting Items for Systematic Reviews and Meta-Analyses
RCTs: Randomized Controlled Trials
RoB 2: Risk of Bias 2 Tool
SMD: Standardized Mean Difference
TNF-alpha: Tumor Necrosis Factor Alpha
WHF: World Heart Federation
