# Supplementary material for: Comparative effects of atorvastatin and rosuvastatin on inflammatory biomarkers: a systematic review and meta-analysis of randomized head-to-head trials
Source: BMC Cardiovasc Disord. 2026 Sep 10;26:786. doi: 10.1186/s12872-026-06386-4 (PMC13560371; doi:10.1186/s12872-026-06386-4)
Supplement: Supplementary file 1 [file 12872_2026_6386_MOESM4_ESM.docx]

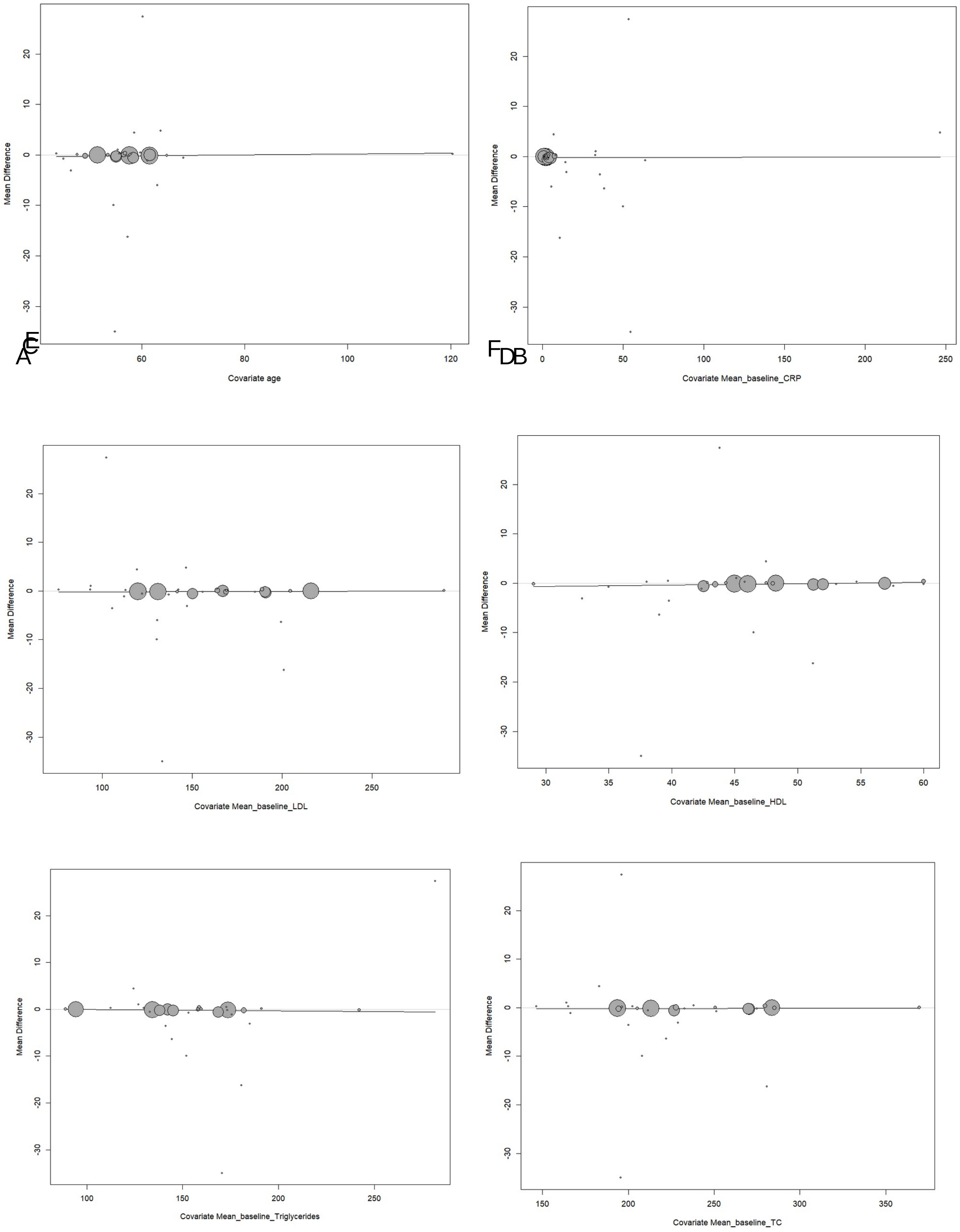

A
C
E
F
D
B
Mean Difference
Covariate age
Covariate Mean_baseline_CRP
Covariate Mean_baseline_LDL
Covariate Mean_baseline_HDL
Covariate Mean_baseline_Triglycerides
Covariate Mean_baseline_TC

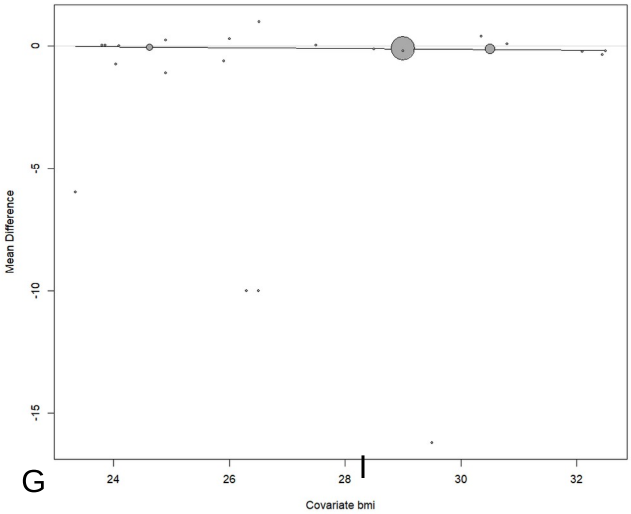
Mean Difference
0
-5
-10
-15
24
26
28
30
32
Covariate bmi

G

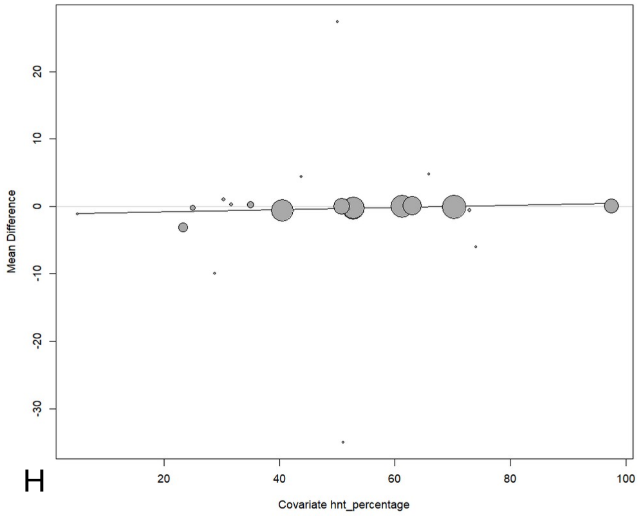
Mean Difference
20
10
0
-10
-20
-30
20
40
60
80
100
Covariate hnt_percentage

H

I

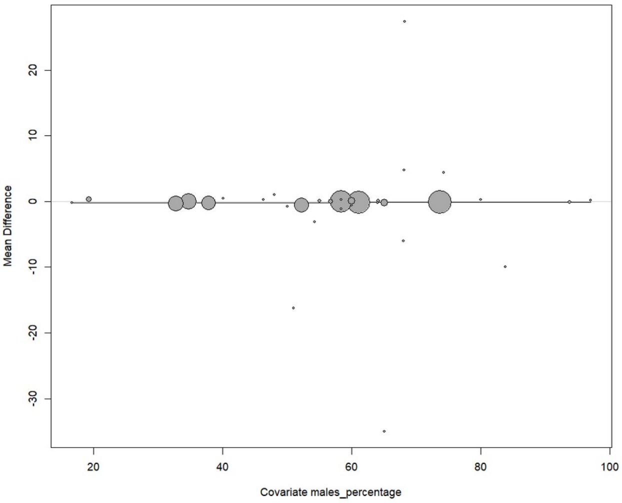
Mean Difference
20
10
0
-10
-20
-30
20
40
60
80
100
Covariate males_percentage
