# Supplementary material for: Comparative effects of atorvastatin and rosuvastatin on inflammatory biomarkers: a systematic review and meta-analysis of randomized head-to-head trials
Source: BMC Cardiovasc Disord. 2026 Sep 10;26:786. doi: 10.1186/s12872-026-06386-4 (PMC13560371; doi:10.1186/s12872-026-06386-4)
Supplement: Supplementary file 2 [file 12872_2026_6386_MOESM5_ESM.docx]

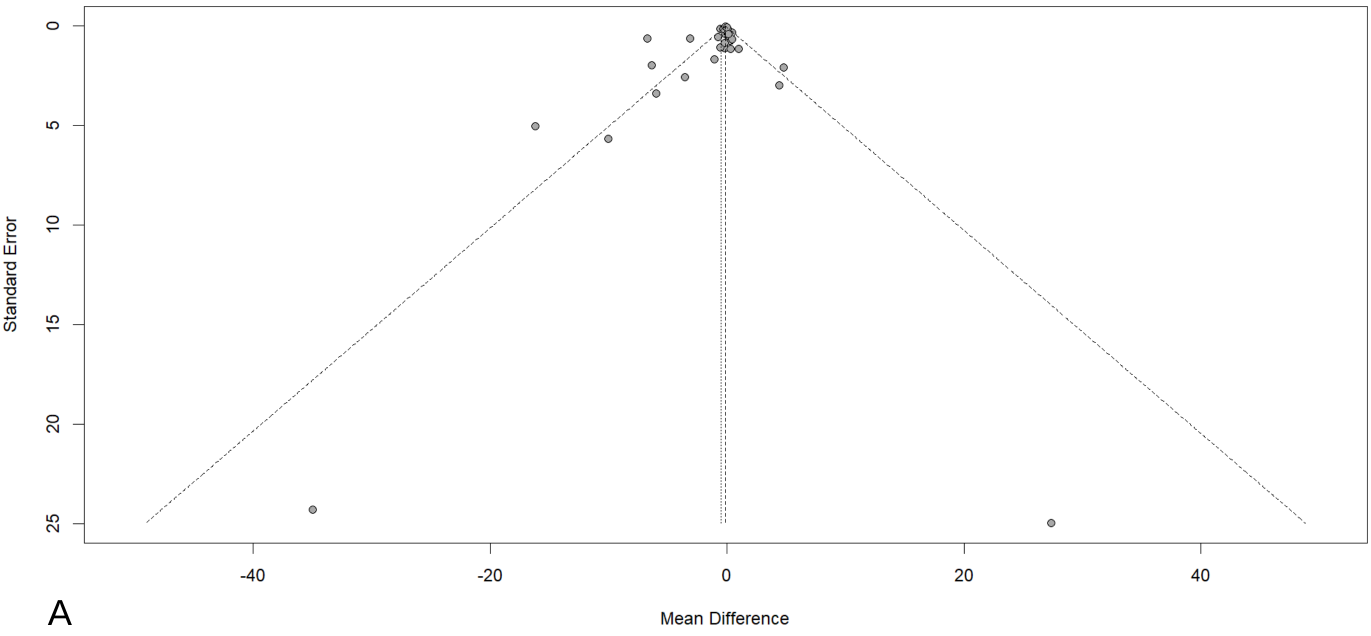

Standard Error
0
5
10
15
20
25
-40
-20
0
20
40
Mean Difference

A

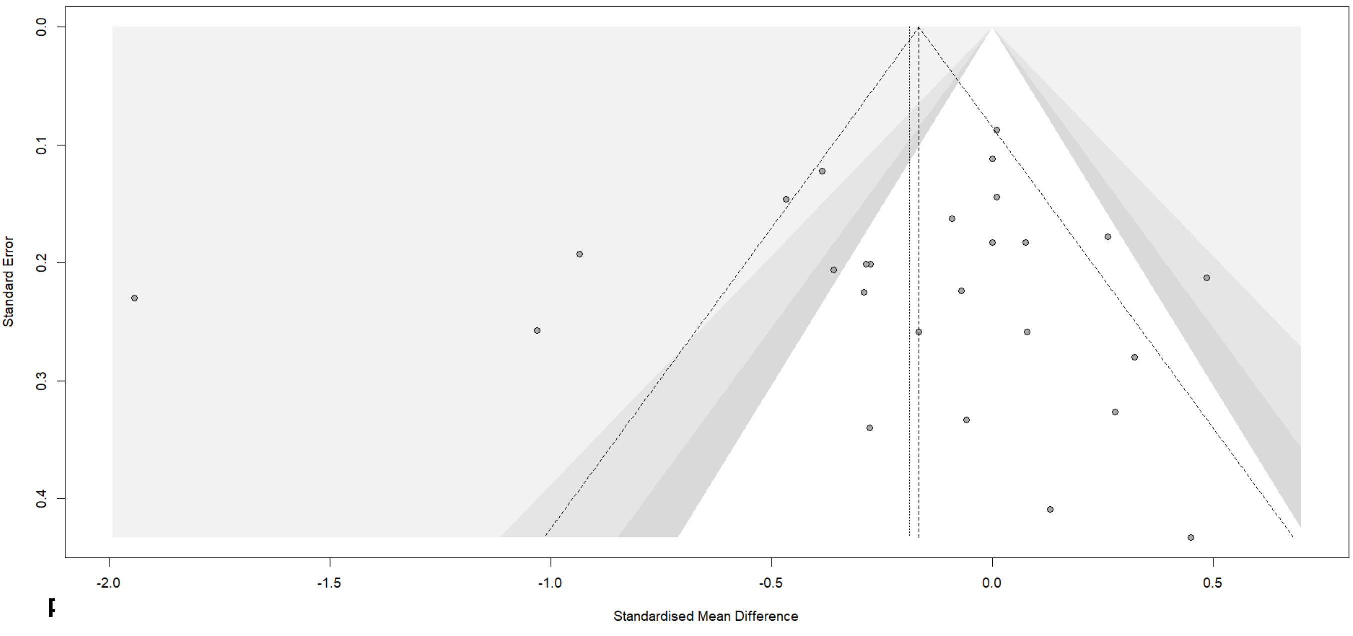

Standard Error
0.0
0.1
0.2
0.3
0.4
-2.0
-1.5
-1.0
-0.5
0.0
0.5
Standardised Mean Difference
