# Supplementary material for: Comparative effects of atorvastatin and rosuvastatin on inflammatory biomarkers: a systematic review and meta-analysis of randomized head-to-head trials
Source: BMC Cardiovasc Disord. 2026 Sep 10;26:786. doi: 10.1186/s12872-026-06386-4 (PMC13560371; doi:10.1186/s12872-026-06386-4)
Supplement: Supplementary file 1 [file 12872_2026_6386_MOESM1_ESM.docx]

**Search strategy:**

| "Atorvastatin"[Mesh]  Lipitor  Atorvastatin Calcium  Atorvastatin, Calcium Salt  Atorvastatin Calcium Anhydrous  Liptonorm  Atorvastatin Calcium Hydrate  CI 981  CI-981  CI981  Atorvastatin Calcium Trihydrate  Atorvastatin* | "Rosuvastatin Calcium"[Mesh]  Calcium, Rosuvastatin  Rosuvastatin  ZD4522  ZD 4522  Crestor  Rosuvastatin* | "Interleukin-6"[Mesh]  Interleukin 6  interleukin-6  IL-6  IL6  interleukin* | "Tumor Necrosis Factor-alpha"[Mesh]  Tumor Necrosis Factor alpha  TNF-alpha  Tumor Necrosis Factor  necrosis factor | "Adiponectin"[Mesh]  Adiponectin  Adiponectin* | "C-Reactive Protein"[Mesh]  CRP  C Reactive Protein  hs-CRP  hsCRP  High Sensitivity C-Reactive Protein  High Sensitivity C Reactive Protein | inflammatory marker*  inflammatory biomarker* |
| --- | --- | --- | --- | --- | --- | --- |

**PUBMED: (**119 records)

("Atorvastatin"[Mesh] OR Lipitor OR "Atorvastatin Calcium" OR "Atorvastatin, Calcium Salt" OR "Atorvastatin Calcium Anhydrous" OR Liptonorm OR "Atorvastatin Calcium Hydrate" OR "Atorvastatin Calcium Trihydrate" OR Atorvastatin*) AND ("Rosuvastatin Calcium"[Mesh] OR "Calcium, Rosuvastatin" OR Rosuvastatin OR Rosuvastatin*) AND ("Interleukin-6"[Mesh] OR "Interleukin 6" OR "interleukin-6" OR IL-6 OR IL6 OR interleukin* OR "Tumor Necrosis Factor-alpha"[Mesh] OR "Tumor Necrosis Factor alpha" OR TNF-alpha OR "Tumor Necrosis Factor" OR "necrosis factor" OR "Adiponectin"[Mesh] OR Adiponectin OR Adiponectin* OR "C-Reactive Protein"[Mesh] OR "C Reactive Protein" OR hs-CRP OR CRP OR hsCRP OR "High Sensitivity C-Reactive Protein" OR "High Sensitivity C Reactive Protein" OR "inflammatory marker*" OR "inflammatory biomarker*")

**Web of science: (**456 records)

("Atorvastatin" OR Lipitor OR "Atorvastatin Calcium" OR "Atorvastatin, Calcium Salt" OR "Atorvastatin Calcium Anhydrous" OR Liptonorm OR "Atorvastatin Calcium Hydrate" OR "Atorvastatin Calcium Trihydrate" OR Atorvastatin*) AND ("Rosuvastatin Calcium" OR "Calcium, Rosuvastatin" OR Rosuvastatin OR Rosuvastatin*) AND ("Interleukin-6" OR "Interleukin 6" OR "interleukin-6" OR IL-6 OR IL6 OR interleukin* OR "Tumor Necrosis Factor-alpha" OR "Tumor Necrosis Factor alpha" OR TNF-alpha OR "Tumor Necrosis Factor" OR "necrosis factor" OR "Adiponectin" OR Adiponectin OR Adiponectin* OR "C-Reactive Protein" OR "C Reactive Protein" OR hs-CRP OR CRP OR hsCRP OR "High Sensitivity C-Reactive Protein" OR "High Sensitivity C Reactive Protein" OR "inflammatory marker" OR "inflammatory biomarker")

**Scopus**: (36 records)

( ALL ( ( "Atorvastatin" OR lipitor OR "Atorvastatin Calcium" OR "Atorvastatin, Calcium Salt" OR "Atorvastatin Calcium Anhydrous" OR liptonorm OR "Atorvastatin Calcium Hydrate" OR "Atorvastatin Calcium Trihydrate" OR atorvastatin* ) ) AND ALL ( ( "Rosuvastatin Calcium" OR "Calcium, Rosuvastatin" OR rosuvastatin OR rosuvastatin* ) ) AND ALL ( ( "Interleukin-6" OR "Interleukin 6" OR "interleukin-6" OR il-6 OR il6 OR interleukin* OR "Tumor Necrosis Factor-alpha" OR "Tumor Necrosis Factor alpha" OR tnf-alpha OR "Tumor Necrosis Factor" OR "necrosis factor" OR "Adiponectin" OR adiponectin OR adiponectin* OR "C-Reactive Protein" OR "C Reactive Protein" OR hs-crp OR crp OR hscrp OR "High Sensitivity C-Reactive Protein" OR "High Sensitivity C Reactive Protein" OR "inflammatory marker" OR "inflammatory biomarker" ) ) )

**Cochrane** (156 records)

("Atorvastatin" OR Lipitor OR "Atorvastatin Calcium" OR "Atorvastatin, Calcium Salt" OR "Atorvastatin Calcium Anhydrous" OR Liptonorm OR "Atorvastatin Calcium Hydrate" OR "Atorvastatin Calcium Trihydrate" OR Atorvastatin*) in All Text AND ("Rosuvastatin Calcium" OR "Calcium, Rosuvastatin" OR Rosuvastatin OR Rosuvastatin*) in All Text AND ("Interleukin-6" OR "Interleukin 6" OR "interleukin-6" OR IL-6 OR IL6 OR interleukin* OR "Tumor Necrosis Factor-alpha" OR "Tumor Necrosis Factor alpha" OR TNF-alpha OR "Tumor Necrosis Factor" OR "necrosis factor" OR "Adiponectin" OR Adiponectin OR Adiponectin* OR "C-Reactive Protein" OR "C Reactive Protein" OR hs-CRP OR CRP OR hsCRP OR "High Sensitivity C-Reactive Protein" OR "High Sensitivity C Reactive Protein" OR "inflammatory marker" OR "inflammatory biomarker") in All Text - (Word variations have been searched)
