# Supplementary material for: Comparative effects of atorvastatin and rosuvastatin on inflammatory biomarkers: a systematic review and meta-analysis of randomized head-to-head trials
Source: BMC Cardiovasc Disord. 2026 Sep 10;26:786. doi: 10.1186/s12872-026-06386-4 (PMC13560371; doi:10.1186/s12872-026-06386-4)
Supplement: Supplementary file 2 [file 12872_2026_6386_MOESM2_ESM.docx]

| **Author Year** | **Intervention** | **Baseline Inflammatory Biomarkers (Mean**±**SD)** | | | | **Baseline Comorbidities(%)** | | | | | **Conclusion** |
| --- | --- | --- | --- | --- | --- | --- | --- | --- | --- | --- | --- |
|  |  | **CRP (mg/L)** | **Adiponectin (pg/ml)** | **IL-6 (μg/ml)** | **TNF-alpha (pg/ml)** | **Previous MI** | **Hypertension** | **Coronary artery disease** | **Cerebrovascular desease** | **Dyslipidemia** |  |
| **Shioji 2014** | **ATV** | 1.2±1.6 | N/A | N/A | N/A | N/A | 79.2 | 72.7 | 9.1 | 100 | RSV is an effective treatment choice for Japanese patients who fail to meet their JAS2007GL LDL-C targets with ATV. |
|  | **RSV** | 1.2±2.4 | N/A | N/A | N/A | N/A | 68 | 69.3 | 10.7 | 100 |  |
| **Sindhu 2011** | **ATV** | 2.78±0.55 | N/A | N/A | N/A | N/A | N/A | N/A | N/A | N/A | This study highlights that RSV is preferable for obese T2DM patients with abnormal LDL-C and TC, while statin benefits hs-CRP, HDL-C, and TG. |
|  | **RSV** | 2.89±0.66 | N/A | N/A | N/A | N/A | N/A | N/A | N/A | N/A |  |
| **Stein 2003** | **ATV** | 2.4±2.8 | N/A | N/A | N/A | N/A | N/A | N/A | N/A | N/A | RSV consistently outperforms atorvastatin in reducing LDL cholesterol and raising HDL cholesterol, achieving optimal lipid levels, especially in high-risk patients with familial hypercholesterolemia. |
|  | **RSV** | 2.3±2.5 | N/A | N/A | N/A | N/A | N/A | N/A | N/A | N/A |  |
| **Thongtan 2011** | **ATV** | 9±17.7 | 19.12±27.6 | N/A | N/A | N/A | N/A | 20.4 | N/A | N/A | Both maximal doses of ATV and RSV effectively lowered LDL-C and triglyceride levels, with RSV being more effective in raising HDL-C. While both statins increased insulin levels and reduced CRP, only atorvastatin significantly raised glycated albumin levels. |
|  | **RSV** | 22.05, 45.2 | 26.97±47.07 | N/A | N/A | N/A | N/A | 19.6 | N/A | N/A |  |
| **Tian 2018** | **ATV** | 7.6±0.83 | N/A | N/A | N/A | 37 | 63 | UA-16,  Stemi-7, NSTEMI-4 | 16.67 | N/A | All the statins help reduce lipid levels and inflammation in atherosclerotic patients. They also show potential renal protective effects, particularly in decreasing serum creatinine and Cystatin C. |
|  | **RSV** | 7.50±0.68 | N/A | N/A | N/A | 40 | 63 | UA-18,STEMI-7, NSTEMI-5 | 13.3 | N/A |  |
| **Tian 2017** | **ATV** | 5.24±2.19 | N/A | 0.0151±0.00479 | N/A | N/A | N/A | N/A | N/A | N/A | RSV was more effective than ATV in lowering LDL-C, inhibiting microinflammation, and improving nutritional status in these patients |
|  | **RSV** | 5.08±1.17 | N/A | 0.0162±0.00429 | N/A | N/A | N/A | N/A | N/A | N/A |  |
| **Tran 2021** | **ATV** | 4.71±5.41 | N/A | N/A | N/A | STEMI-47.9,NSTEMI-52.1 | 81.3 | 100 | N/A | 54.2 | RSV is more effective  than ATV in achieving LDL-c targets of <1.8 mmol/L after 4 days in Vietnamese patients with ACS. |
|  | **RSV** | 7.72±10.25 | N/A | N/A | N/A | STEMI- 45.8,NSTEMI- 54.2 | 66.7 | 100 | N/A | 52.1 |  |
| **Umrani 2020** | **ATV** | 38.79±11.28 | N/A | N/A | N/A | N/A | N/A | 100 | N/A | N/A | RSV was significantly more effective than ATV in reducing inflammatory markers like ESR and hsCRP in patients with ACS. Cardiologists should consider using rosuvastatin over atorvastatin for managing ACS patients with elevated inflammatory biomarkers. |
|  | **RSV** | 39.34±10.29 | N/A | N/A | N/A | N/A | N/A | 100 | N/A | N/A |  |
| **Anagnostis P et al. 2011** | **ATV** | 3.1±2.3 | 10.2±3.9 | N/A | N/A | N/A | N/A | N/A | N/A | 100 | RSV resulted in significant improvement in insulin resistance. On the other hand, the other parameters showed no significant change. |
|  | **RSV** | 2.8±2.5 | 16.5±11.1 | N/A | N/A | N/A | N/A | N/A | N/A | 100 |  |
| **Anagnostis P et al. 2014** | **ATV** | 4.1±1.4 | N/A | N/A | N/A | N/A | N/A | N/A | N/A | 100 | ATV and RSV did not significantly affect 25(OH)D levels |
|  | **RSV** | 2.8±0.5 | N/A | N/A | N/A | N/A | N/A | N/A | N/A | 100 |  |
| **Aydin 2015** | **ATV** | 8.2±3 | N/A | 0.04±0.025 | N/A | 100 | 39 | N/A | N/A | N/A | RSV was more effective in increasing HDL-C. |
|  | **RSV** | 8.1±3.1 | N/A | 0.039±0.027 | N/A | 100 | 31 | N/A | N/A | N/A |  |
| **Brunetti 2007** | **ATV** | 75.3±74.6 | N/A | N/A | N/A | 9.1 | 45.5 | N/A | N/A | N/A | 3-month treatment with either ATV 20 mg or 10 mg RSV is effective in improving endothelium dysfunction in patients with diabetes. |
|  | **RSV** | 31.8±24.3 | N/A | N/A | N/A | 0 | 54.5 | N/A | N/A | N/A |  |
| **Cao 2017** | **ATV** | 14.87±2.97 | N/A | 0.1399±0.0169 | 185.74±27.41 | N/A | N/A | N/A | 100 | N/A | Statin treatment has no significance on the antiplatelet function of clopidogrel. RSV shows better control of blood lipids, carotid atherosclerosis, and inflammatory factors. |
|  | **RSV** | 15.11±3.07 | N/A | 0.1406±0.0175 | 190.17±26.33 | N/A | N/A | N/A | 100 | N/A |  |
| **Cimato 2015** | **ATV** | 1.05±1.1 | N/A | N/A | N/A | N/A | N/A | N/A | N/A | N/A | Total cholesterol levels positively regulate IL-8, while HDL cholesterol and apolipoprotein A1 reduces IL-12 p40 and IL-18 binding protein levels. Additional studies need to be made to verify these findings. |
|  | **RSV** | 1.3±1.5 | N/A | N/A | N/A | N/A | N/A | N/A | N/A | N/A |  |
| **Darban 2021** | **ATV** | 9.8±5.1 | N/A | N/A | N/A | N/A | 6.8 | 33.3 | N/A | N/A | High-dose therapies with ATV and RSV have similar effects on lipid profiles and hs-CRP levels in patients undergoing PCI |
|  | **RSV** | 10±6.7 | N/A | N/A | N/A | N/A | 3.8 | 33.3 | N/A | N/A |  |
| **Said 2021** | **ATV** | N/A | N/A | 27.3±17.41 | N/A | N/A | 66.7 | N/A | N/A | 14.3 | Lipophilic ATV is superior to hydrophilic RSV in increasing left ventricular ejection fraction and reducing fibrosis marker sST2 in HF patients. |
|  | **RSV** | N/A | N/A | 24.1±14.63 | N/A | N/A | 55.8 | N/A | N/A | 11.6 |  |
| **Gorji 2023^1^** | **ATV** | 38.9±16.82 | N/A | 0.00000327±0.0000016 | N/A | N/A | 26.3 | N/A | N/A | N/A | Moderate-intensity statins are not adequate for the majority of patients with T2DM and mild hyperlipidemia. Greater numbers of patients could reach LDL targets with high-intensity statin therapy. |
|  | **RSV** | 27.5±25.6 | N/A | 0.00000329±0.00000136 | N/A | N/A | 31.6 | N/A | N/A | N/A |  |
| **Gorji 2023^2^** | **ATV** | 35.23±31 | N/A | 0.00000323±0.00000175 | N/A | N/A | 33.3 | N/A | N/A | N/A |  |
|  | **RSV** | 31±32.7 | N/A | 0.00000331±0.00000168 | N/A | N/A | 31.6 | N/A | N/A | N/A |  |
| **Hong 2011** | **ATV** | 9.3±20.3 | N/A | N/A | N/A | UA:33,52  NSTEMI:16,26  STEMI:5,8 | 44 | N/A | N/A | N/A | Moderate doses of RSV and ATV could contribute to plaque regression. |
|  | **RSV** | 5±11.7 | N/A | N/A | N/A | UA:29,45  NSTEMI:5,8  STEMI:14,22 | 43 | N/A | N/A | N/A |  |
| **Karalis 2010** | **ATV** | 2.5±1.8 | N/A | N/A | N/A | N/A | N/A | N/A | N/A | N/A | RSV and ATV displayed similar patterns of effect on lipid metabolism. |
|  | **RSV** | 2.5±1.9 | N/A | N/A | N/A | N/A | N/A | N/A | N/A | N/A |  |
| **SAKU2011** | **ATV** | 11±23 | N/A | N/A | N/A | 1 | 64.6 | 8.1 | 6.1 | N/A | The safety and efficacy of the 3 statins are equal. The 3 statins should be used at the complete discretion of the physician based on the patient’s background |
|  | **RSV** | 11±18 | N/A | N/A | N/A | 3 | 58 | 8.1 | 6.1 | N/A |  |
| **Khurana 2015** | **ATV** | 35.48±11.65 | N/A | N/A | N/A | N/A | N/A | 100 | N/A | N/A | Both ATV (40 mg) and RSV (20 mg) are effective in decreasing CRP and LDL in about 4 weeks, RSV proved more efficient in decreasing CRP |
|  | **RSV** | 35.88±9.87 | N/A | N/A | N/A | N/A | N/A | 100 | N/A | N/A |  |
| **Kumar 2019** | **ATV** | 38.19 ± 12.38 | N/A | N/A | N/A | N/A | N/A | 100 | N/A | N/A | RSV showed a 50% decrease and ATV showed a 35% reduction hs-CRP levels in  ACS patients. RSV is more effective in reducing micro-inflammation in ACS  patients |
|  | **RSV** | 38.34±10.23 | N/A | N/A | N/A | N/A | N/A | 100 | N/A | N/A |  |
| **Milionis 2006** | **ATV** | 3.3 | N/A | N/A | N/A | N/A | 50 | N/A | N/A | N/A | RSV and ATV were equally  efficacious in achieving LDL-C  In primary hyperlipidemia  RSV raises HDL significantly,  while ATV lowers serum uric acid levels |
|  | **RSV** | 3.1 | N/A | N/A | N/A | N/A | 51.7 | N/A | N/A | N/A |  |
| **Mostafa 2018** | **ATV** | 37.4±73.2 | N/A | N/A | N/A | STEMI 82, NSTEMI 17,UA20 | 52 | N/A | N/A | N/A | RSV 40 mg/day is safer and more effective than ATV 80 mg/day in terms of  lipid parameters and inflammatory biomarkers |
|  | **RSV** | 72±147 | N/A | N/A | N/A | STEMI 56, NSTEMI 34, UA30 | 50 | N/A | N/A | N/A |  |
| **Namal 2011** | **ATV** | 27.3±11.7 | N/A | N/A | N/A | N/A | 64.4 | N/A | N/A | N/A | high-dose lipid-lowering agent ezetimibe/simvastatin 10 mg/day combination therapy initiated early in patients  presenting with unstable angina and non-ST segment elevation myocardial infarction is superior to ATV 20 mg/day and RSV 10 mg/day in terms of  reducing inflammatory markers |
|  | **RSV** | 22.0±6.9 | N/A | N/A | N/A | N/A | 67.4 | N/A | N/A | N/A |  |
| **Qu 2009** | **ATV** | 3.3±0.4 | 11.74±7.8 | N/A | 3.45±0.42 | N/A | N/A | N/A | N/A | N/A | Therapy with both statins  decreased  levels of vascular biomarkers hs-CRP, MMP-9, and PAI-1  But only RSV increased serum  adiponectin levels significantly in patients with hypercholesterolemia, which is beneficial  in CAD |
|  | **RSV** | 3.5±0.5 | 9.82±5.71 | N/A | 3.21±0.52 | N/A | N/A | N/A | N/A | N/A |  |
| **Tsutamoto 2011** | **ATV** | 0.91±0.56 | N/A | N/A | N/A | N/A | N/A | N/A | N/A | 9 | Lipophilic ATV but not hydrophilic RSV improves cardiac sympathetic nerve activity in CHF patients with DCM |
|  | **RSV** | 0.68,±0.15 | N/A | N/A | N/A | N/A | N/A | N/A | N/A | 16 |  |
| **Elhadad 2024** | **ATV** | 47±25 | N/A | N/A | N/A | 100 | 27.5 | 100 | N/A | N/A | High-intensity RSV was superior to high-intensity ATV reducing the inflammatory response and myocardial fibrosis, thus improving ventricular remodeling and cardiac function better in STEMI patients. |
|  | **RSV** | 53±23 | N/A | N/A | N/A | 100 | 30 | 100 | N/A | N/A |  |
| **Betteridge 2007** | **ATV** | 2.24±2.088 | N/A | N/A | N/A | N/A | N/A | N/A | N/A | N/A | CRP was decreased in patients with type 2 diabetes recieving RSV or atorvastatin. RSV decreased LDL cholesterol more significantly than ATV. |
|  | **RSV** | 2.38±2.23 | N/A | N/A | N/A | N/A | N/A | N/A | N/A | N/A |  |
| **Nicholls 2011** | **ATV** | 1.9±1.86 | N/A | N/A | N/A | 26.4 | 70.7 | N/A | N/A | N/A | Maximal doses of RSV and ATV led to significant regression of coronary atherosclerosis. Although RSV achieved lower LDL cholesterol and higher HDL cholesterol levels, both treatments resulted in a similar degree of regression of peripheral arterial vascular (PAV) disease. |
|  | **RSV** | 2.12±2.23 | N/A | N/A | N/A | 22.5 | 70 | N/A | N/A | N/A |  |
| **Liu B et al 2011** | **ATV** | 1.9±4.22 | N/A | N/A | N/A | N/A | 22.2 | 50 | 11.1 | N/A | Both statins significantly reduced total cholesterol, LDL-C, triglycerides, and hsCRP after 4 weeks, with no significant differences between the two. However, RSV caused a greater reduction in ROCK activity and a larger improvement in endothelial function (FMD). |
|  | **RSV** | 2.2±3.82 | N/A | N/A | N/A | N/A | 27.8 | 55.6 | 11.1 | N/A |  |
| **Ferdinand 2006^1^** | **ATV** | 4.22±3.81 | N/A | N/A | N/A | N/A | N/A | N/A | N/A | N/A | RSV 10 mg and 20 mg showed greater effectiveness in improving the lipid profile of hypercholesterolemic African-Americans compared to equivalent doses of ATV, highlighting its superior lipid-lowering capability. |
|  | **RSV** | 4.07±4026 | N/A | N/A | N/A | N/A | N/A | N/A | N/A | N/A |  |
| **Ferdinand 2006^2^** | **ATV** | 5.07±3.14 | N/A | N/A | N/A | N/A | N/A | N/A | N/A | N/A |  |
|  | **RSV** | 5.01±4.33 | N/A | N/A | N/A | N/A | N/A | N/A | N/A | N/A |  |
| **Park 2010** | **ATV** | 2.23± 0.35 | N/A | N/A | N/A | N/A | N/A | N/A | N/A | N/A | In patients with nondiabetic metabolic syndrome, RSV 10 mg was superior to ATV 10 mg in reaching the NCEP ATP III LDL-C targets. |
|  | **RSV** | 2.29+/- 0.30 | N/A | N/A | N/A | N/A | N/A | N/A | N/A | N/A |  |
| **Puccetti 2011** | **ATV** | N/A | N/A | N/A | N/A | N/A | N/A | N/A | N/A | N/A | Both ATV and RSV cause reduction of thromboxane-dependent platelet activation, lipid peroxidation and inflammation. |
|  | **RSV** | N/A | N/A | N/A | N/A | N/A | N/A | N/A | N/A | N/A |  |
| **Stalenhoef 2005** | **ATV** | 2.8 (0.7-8.2) ( median[10th-90th percentile]) | N/A | N/A | N/A | N/A | N/A | N/A | N/A | N/A | RSV was more effective than ATV in lowering LDL-C and improving the lipid profile, with better tolerance in metabolic syndrome patients. |
|  | **RSV** | 2.3 (0.9-8.1) ( median[10th-90th percentile]) | N/A | N/A | N/A | N/A | N/A | N/A | N/A | N/A |  |

**ATV**, Atorvastatin; **RSV**, Rosuvastatin; **LDL**, Low-Density Lipoprotein; **HDL**, High-Density Lipoprotein; **TG**, Triglycerides; **TC**, Total Cholesterol; **CRP**, C-Reactive Protein; **IL-6**, Interleukin-6; **TNF-alpha**, Tumor Necrosis Factor-Alpha; **MRAs**, Mineralocorticoid Receptor Antagonists; **ACE-I**, Angiotensin-Converting Enzyme Inhibitors; **ARBs**, Angiotensin Receptor Blockers; **β-blockers**, Beta-Adrenergic Blockers; **Ca channel blockers**, Calcium Channel Blockers; **ACS**, Acute Coronary Syndrome; **MI**, Myocardial Infarction; **HF**, Heart Failure; **UA**, Unstable Angina; **STEMI**, ST-Elevation Myocardial Infarction; **NSTEMI**, Non-ST-Elevation Myocardial Infarction; **PCI**, Percutaneous Coronary Intervention; **FMD**, Flow-Mediated Dilatation; **CHF**, Congestive Heart Failure; **Type 2 DM**, Type 2 Diabetes Mellitus; **MMP-9**, Matrix Metalloproteinase-9; **PAI-1**, Plasminogen Activator Inhibitor-1; **sST2**, Soluble Suppression of Tumorigenicity 2 (fibrosis marker).

**Ferdinand 2006¹ –** Refers to a group of patients receiving atorvastatin 10 mg and rosuvastatin 10 mg.

**Ferdinand 2006² –** Refers to a group of patients receiving atorvastatin 20 mg and rosuvastatin 20 mg**.**

**Gorji 2023¹ –** Refers to a group of patients receiving atorvastatin 40 mg and rosuvastatin 20 mg.

**Gorji 2023² –** Refers to a group of patients receiving atorvastatin 20 mg and rosuvastatin 10 mg.
