# Supplementary material for: Comparative effects of atorvastatin and rosuvastatin on inflammatory biomarkers: a systematic review and meta-analysis of randomized head-to-head trials
Source: BMC Cardiovasc Disord. 2026 Sep 10;26:786. doi: 10.1186/s12872-026-06386-4 (PMC13560371; doi:10.1186/s12872-026-06386-4)
Supplement: Supplementary file 8 [file 12872_2026_6386_MOESM3_ESM.docx]

(A)
(B)
(C)
(D)
